# Self-assembled magnesium peroxide supramolecular hydrogel for oxidants neutralization and chemical burn management

**DOI:** 10.1016/j.bioactmat.2025.11.002

**Published:** 2025-11-12

**Authors:** Meng Zhang, Wanting Hao, Zi Fu, Ying Huang, Fuhua Yan, Dalong Ni

**Affiliations:** a Department of Orthopaedics, Shanghai Key Laboratory for Prevention and Treatment of Bone and Joint Diseases, Shanghai Institute of Traumatology and Orthopaedics, Ruijin Hospital, Shanghai Jiao Tong University School of Medicine, Shanghai, 200025, PR China; b Department of Radiology, Ruijin Hospital, Shanghai Jiao Tong University School of Medicine, NO. 197 Ruijin Er Road, Shanghai, 200025, PR China; c Faculty of Medical Imaging Technology, College of Health Science and Technology, Shanghai Jiao Tong University School of Medicine, PR China

**Keywords:** Magnesium peroxide, Hydrogel, Oxygen, Nanomedicine, Burn wound

## Abstract

Supramolecular hydrogels have emerged as a transformative strategy for burn wound management due to their dynamic adaptability and bio-interfacial responses. Herein, a self-assembled magnesium peroxide (MPO) supramolecular hydrogel was engineered as a first-aid intervention for oxidant-induced chemical burns. Unlike conventional approaches that mechanically entrap pre-synthesized MPO nanoparticles within hydrogel matrices, the designed hydrogel system utilized metastable MPO species generated *in situ* during the one-pot synthesis to form a crosslinker-free network through hydrogen bonding and Mg^2+^ coordination. The MPO species dynamically assembled into the hydrogel matrix by serving as structural nodes, while the tailored linkers dictated mechanical robustness. The hydrogel exhibited superior efficacy to effectively neutralize residual oxidants by chemical burn (e.g., NaClO), while sustained oxygen release and Mg^2+^ supply from MPO synergistically established a regenerative microenvironment to accelerate angiogenesis and epithelialization. As validated on a murine NaClO-induced chemical burn model, the developed hydrogel achieved excellent oxidant clearance and reduced wound area compared to controls, demonstrating its efficacy as an emergency treatment. This work pioneers a novel hydrogel architecture integrating active MPO species, offering a blueprint for designing oxygen-supplying biomaterials as next-generation therapeutic hydrogels in emergency trauma care.

## Highlights

• A self-assembled MPO-based supramolecular hydrogel was fabricated.
• The hydrogel was formed through hydrogen bonding and metal ion coordination between MPO species and arginine.
• The hydrogel showed a controlled release of hydrogen peroxide to neutralize oxidants.
• The hydrogel presented the potential for in-time oxidant-induced chemical burn treatment.

## Graphical abstract

**Figure:**
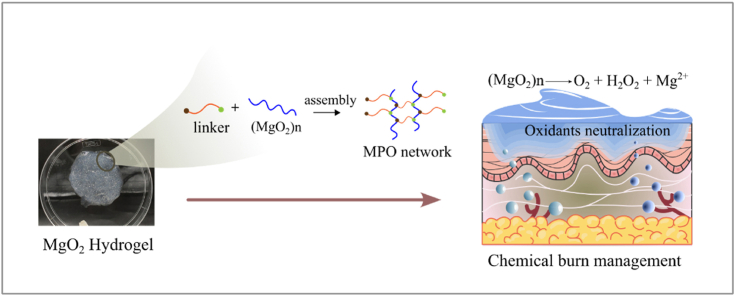

## 1 Introduction

Hydrogels with their excellent biocompatibility and flexibility have shown great potential in biomedical applications such as drug delivery, tissue engineering and wound healing [1,2]. However, while significant progress has been made in addressing various injuries [3,4], the treatment of chemical burns presents a unique and complex challenge [5]. Unlike heat burns that primarily affect the skin's surface, chemical burns which are caused by strong acids, bases, or oxidizing agents, penetrate deeply into the skin and underlying tissues, making them extremely difficult to treat [6]. Current medical guidelines, such as rinsing with extensive water, are often fall short in removing harmful substances that have already been absorbed [7]. Additionally, general neutralizing agents like sodium bicarbonate or boric acid as studied in emergency treatments, may be ineffective or even worsen tissue damages, leading to secondary deep burns [8]. Given these challenges, effective treatment of chemical burns requires not only the neutralization of harmful chemicals but also the further wound healing promotion. This necessitates the development of comprehensive treatment hydrogels with both gentle neutralization and wound healing management for chemical burn injuries.

Recently, magnesium peroxide (MPO) has emerged as a promising candidate in the biomedical field due to its ability to slowly release magnesium ions (Mg^2+^), oxygen, and hydrogen peroxide (H_2_O_2_) in aqueous environments, offering distinct advantages over other peroxide-based biomaterials [[9], [10], [11], [12]]. Compared to the most widely studied calcium peroxide (CPO), MPO hydrolysis yields Mg(OH)_2_, resulting in a moderate pH increase (typically <8.5), thereby maintaining near-physiological conditions in wounds. In contrast, CPO generates Ca(OH)_2_ and elevates pH to over 9, which risks protein denaturation and delayed healing. Moreover, the released Mg^2+^ demonstrate greater angiogenic potential and anti-inflammatory effects, while posing no risk of hypercalcemia at therapeutic doses [[13], [14], [15], [16]]. Unlike unstable alternatives such as urea peroxide or rapidly dissolving sodium percarbonate, MPO exhibits solid-state stability, facilitating its integration into biomedical scaffolds. These attributes collectively endow MPO with considerable potential for wound healing and infection control [17]. Notably, H_2_O_2_, with its excellent skin permeability, can effectively penetrate under the skin and neutralize highly oxidative chemicals while yield only non-toxic byproducts, namely water and oxygen. This makes MPO an ideal candidate for use as a first-aid neutralizer for burns caused by oxidizing agents. Additionally, the oxygen generated during this process can enhance wound healing by promoting angiogenesis and cellular metabolism [18], while Mg^2+^ have been shown to play a crucial role in tissue regeneration and anti-inflammatory responses [19]. However, despite these advantages, most reported MPO-based hydrogel systems have relied on crosslinking methods, with MPO merely as a payload in the hydrogel matrix [20,21]. This approach failed to fully exploit the potential of MPO in achieving effective chemical burn treatment, as it restricts the controlled release of active components and limits the hydrogel's responsiveness to the wound environment.

Supramolecular hydrogels, which are formed through non-covalent interactions such as hydrogen bonding, metal ion coordination, and electrostatic attraction, offer an alternative strategy [22,23]. These systems allow for the integration of functional components that not only contribute to the three-dimensional network construction but also act as active payloads, enabling precise design and seamless integration of functionality into the gel matrix [[24], [25], [26]]. Reports have demonstrated the direct self-assembly of rhein into supramolecular hydrogel via intermolecular π-π interactions and hydrogen bonds [27]. Recent research from our group also presented a calcium peroxide-based hydrogel, where calcium peroxide units were used as building blocks to cross-link alginate macromolecules and thus forming a robust gel matrix [28]. In this way, calcium peroxide not only reinforced the structural integrity of the gel, but also functioned as antibacterial agents. Herein, we aim to develop MPO-assembled hydrogels that could offer precise control release of active agents and improved tissue healing for the treatment of chemical burns.

**Scheme 1:**
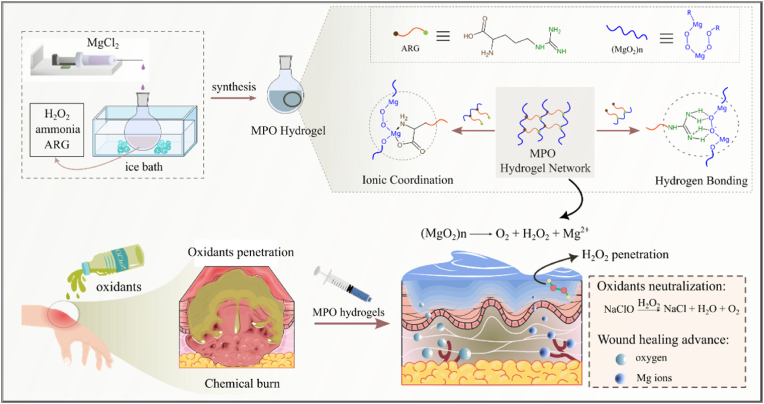
The synthesis of self-assembled MPO supramolecular hydrogel and their application in the first-aid treatment of oxidant-induced chemical burns.

In this work, a self-assembled, crosslinker-free, and highly tunable MPO-based supramolecular hydrogel was reported, and its potential application in the treatment of chemical burns were fully explored. The hydrogel was formed through supramolecular self-assembly of MPO, facilitated by hydrogen bonding, metal ion coordination, and electrostatic attraction between MPO and linker molecules, creating a stable, crosslinker-free three-dimensional network. Notably, the self-assembly behavior heavily relies on the MPO species formed during the one-pot synthesis process, which is critical to their gelation. Direct mixing of pre-synthesized MPO nanoparticles with linker molecules could not lead to hydrogel formation. This state-of-the-art hydrogel exhibited remarkable properties, including structural stability, reshapeability into various forms, excellent biocompatibility and degradability, and controlled release capabilities. In a sodium hypochlorite (NaClO)-induced chemical burn model, the fabricated hydrogel demonstrated a controlled release of hydrogen peroxide, which actively scavenged surface residuals and further neutralized penetrated NaClO within the skin. As a result, the hydrogel effectively deactivated the harmful chemicals and prevented their further diffusion into deeper tissues. Additionally, this process accompanied with the *in situ* generation of oxygen. The localized oxygen supplement and Mg^2+^ further promoted cell growth and accelerated wound healing, offering a comprehensive treatment for chemical burn. Both *in vitro* and *in vivo* studies have demonstrated significant potential of the hydrogel for first-aid application (Scheme 1). This advancement in MPO-based supramolecular hydrogel offers innovative approaches in self-healing materials and tissue regeneration, and further presents a promising "one-stop" solution for chemical burn treatment.

## 2 Results

### 2.1 Characterization of MPO hydrogels

**Fig. 1:**
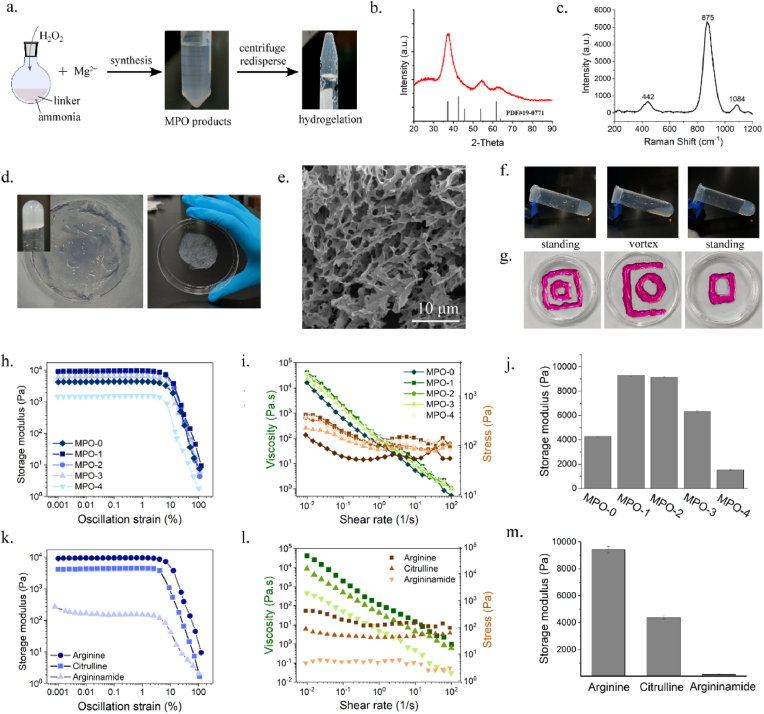
Synthesis and characterization of self-assembled magnesium peroxide (MPO) supramolecular hydrogels. (a) Synthesis illustration of MPO hydrogels. (b) XRD pattern of freeze-dried MPO powder. (c) Raman spectroscopy of freeze-dried MPO powder. (d) Photos of the appearance of MPO supramolecular hydrogels. (e) SEM image of MPO hydrogel. (f) MPO hydrogel transitioned into fluid state while vortexing, and reverted to hydrogel state upon standing. (g) MPO hydrogel (dyed with a red dye) were arranged into various shapes by syringe extrusion. (h) Plots of G’ (storage moduli) versus oscillation strain for MPO hydrogels fabricated using varied amount of arginine. (i) Plots of viscosity and stress versus shear rate for MPO hydrogels fabricated using varied amount of arginine. (j) G′ value of MPO-0, MPO-1, MPO-2, MPO-3 and MPO-4 at 0.1 % of oscillation strain. (k) Plots of G′ versus oscillation strain for MPO hydrogels prepared with arginine, citrulline or argininamide. (l) Plots of viscosity and stress versus shear rate for MPO hydrogels prepared with arginine, citrulline or argininamide. (m) G′ value of MPO hydrogels prepared with arginine, citrulline or argininamide at 0.1 % of oscillation strain.

MPO was prepared through a wet-chemistry method (Fig. 1a). The product was freeze-dried to identify the phase. The X-ray diffraction (XRD) pattern showed three broad peaks at 36.99°, 54.24° and 62.01°, corresponding to the standard diffraction peaks of magnesium peroxide (PDF #19–0771), which conformed the successful formation of magnesium peroxide (Fig. 1b) [29]. Fourier-transform infrared (FT-IR) spectroscopy showed strong peaks at 3442 cm^−1^ and 1636 cm^−1^, attributed to the stretching vibrations of -OH groups and the antisymmetric carboxylate stretching vibration, respectively, indicating the presence of arginine (Fig. S1). Peaks at 1385 cm^−1^ and 1090 cm^−1^ were associated with the symmetric stretching vibration and asymmetric stretching vibration of -O-O-, respectively. Additionally, Raman spectroscopy revealed a strong peak at 875 cm^−1^, signifying the presence of -O-O- groups and further corroborating the dominance of magnesium peroxide (Fig. 1c) [30,31]. Peak at 442 cm^−1^ was assigned to Mg-O lattice vibrations, while the peak at 1084 cm^−1^ potentially indicated the asymmetric stretching vibration of -O-O-. X-ray photoelectron spectroscopy (XPS) analysis confirmed the presence of Mg, O and N (Fig. S2a). The O1s spectrum exhibited binding energy at 532.41 eV and 531.31 eV, which were attributed to O-O bonds and lattice oxygen bonded to Mg, respectively (Fig. S2b). Additionally, the binding energies at 50.37 eV and 49.50 eV indicated the presence of Mg-O and Mg-OH bonds, respectively (Fig. S2c). Simultaneous thermal analysis (STA) revealed three distinct stages of mass changes (Fig. S3). From room temperature to 135 °C, the sample exhibited a 12 % mass loss, attributed to the evaporation of adsorbed water and volatile substances. Between 135 °C and 390 °C, the sample mass showed a gradual decline, accompanied by a sharp exothermic peak near 320 °C, indicating a crystalline phase transformation. Finally, at 390 °C, the sample mass decreased sharply by 27 %, with a significant endothermic peak observed around 422 °C, suggesting that this stage involved the decomposition of the sample with oxygen release. All the results suggested the successful synthesis of MPO.

Intriguingly, while the fresh-prepared MPO was distributed in water, they autonomously self-assembled to form hydrogels (Fig. 1d). The hydrogels exhibited optical transparency or translucency, cream-like texture with low structural strength, and generated numerous bubbles within the structure over several hours, presumably due to oxygen release (Fig. S4). These bubbles could be removed under ultrasound or vortexing, after which the gel structure remained intact. Dilution of the gels with water beyond a certain concentration transformed them into a transparent solution (Fig. S5). Scanning electron microscopy (SEM) observed an interconnected porous architecture of hydrogel (Fig. 1e), while Transmission electron microscopy coupled with energy dispersive x-ray spectroscopy (TEM-EDS) analysis confirmed the presence of O and Mg, with an atomic ratio of 1.75:1 (Fig. S6). The hydrogels exhibited excellent thixotropic behavior, transitioning into a fluid state under high shear forces (e.g. vigorous stirring), and reverting to a hydrogel state upon standing (Fig. 1f). Furthermore, the hydrogels demonstrated remarkable injectability, allowing them to be arranged into various shapes through extrusion using a syringe (Fig. 1g). Oscillatory recovery tests revealed marked recovery of the hydrogel following exposure to cyclic high-to-low shear strains, validating the rapid self-healing behavior essential for *in situ* remodeling post-injection (Fig. S7).

To investigate the origin of hydrogel formation, MPO was prepared by varied ratios of raw materials. The results indicated that the amount of arginine significantly influenced the hydrogelation behavior of MPO. As shown in Fig. 1h, rheological tests were performed on MPO products synthesized with deceasing amount of arginine (denoted as MPO-0, MPO-1, MPO-2, MPO-3, MPO-4) (Fig. S8a). The amplitude sweep curves demonstrated a typical hydrogel behavior: within the low-oscillation strain range, the hydrogels exhibited linear viscoelastic behavior, as the storage modulus (G′) and loss modulus (G″) remaining nearly constant (Fig. S8b). Beyond the critical oscillation strain, both G′ and G″ decreased significantly with increasing strain amplitude. Besides, the flow sweep results indicated obvious shear thinning behavior (Fig. 1i), where viscosity dropped 10^4^-fold as shear rate increased from 0.01 to 100 s^−1^, enabling smooth extrusion through needles.

When the amount of arginine was reduced by 3.5-fold, the G′ value of the formed hydrogels decreased by 6-fold, signifying the critical role of arginine in hydrogelation (Fig. 1j, Fig. S8c). To further elucidate the functional role of arginine, citrulline, argininamide and arginine methyl ester, as structural analogues of arginine, were substituted for arginine in equimolar amounts for MPO hydrogels preparation (Figure S9a, Figure S10a). While both citrulline and argininamide supported hydrogel formation, the resulting hydrogel were weaker than those formed with arginine (Figure S9b, Fig. 1k–m). The G’ values under low oscillation strain decreased by 2-fold and 62-fold for citrulline and argininamide, respectively (Fig. 1m, Fig. S9c–d). In contrast, arginine methyl ester, an analogue with a blocked carboxyl group, completely failed to form a stable hydrogel (Fig. S10), underscoring the critical role of the free carboxylate group in facilitating network cross-linking. These results indicate that the molecular structure of arginine plays a key role in the hydrogelation of MPO.

### 2.2 Mechanism of MPO hydrogel formation

The above results clearly indicated the formation of supramolecular hydrogels, in which the hydrogel network was established through reversible non-covalent interactions. MPO was the key components responsible for network construction. Dynamic light scattering (DLS) analysis revealed ultrasmall diameters for MPO in solution (Fig. S11), and transmission electron microscopy (TEM) images showed a short fibrous morphology (Fig. S12). Selected-area electron diffraction (SAED) analysis further indicated an amorphous state of MPO (Fig. S13), suggesting a self-assembly process of molecular MPO species rather than MPO nanocrystals. Unfortunately, compared to MPO nanocrystals, molecular MPO in solution was much less unstable, particularly upon thermal or hydrolytic conditions. It tended to convert to magnesium hydroxide while releasing oxygen, which complicated further investigations into properties and behavior.

**Fig. 2:**
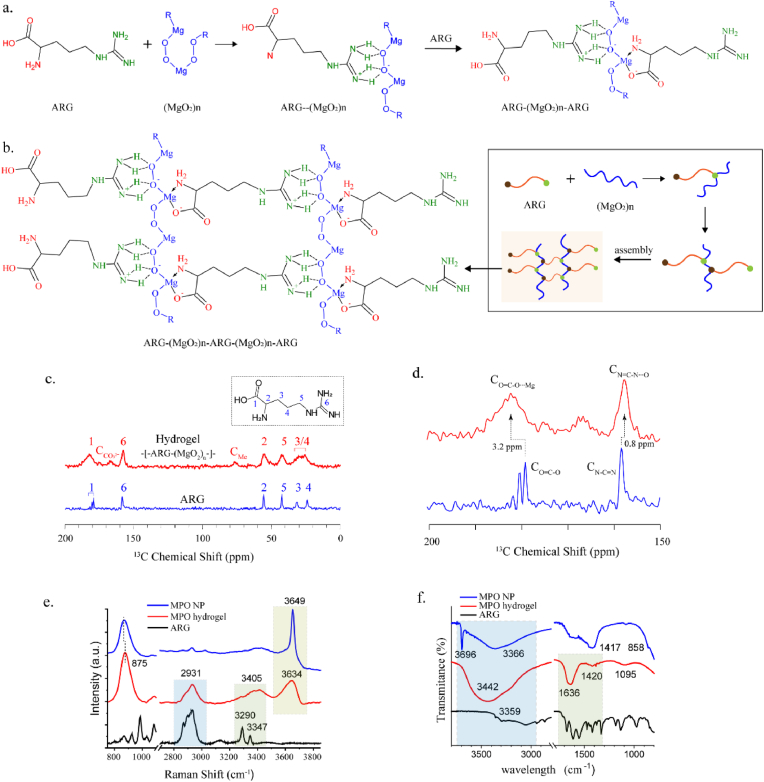
Hypothetical mechanism of MPO hydrogel formation. (a) Hydrogen bonding and ion coordination between MPO and arginine (ARG). (b) Three-dimensional network formation with the interaction of MPO and ARG. (c) ^13^C CP-TOSS NMR spectra of MPO hydrogel, ARG, and molecular structure of arginine in the inset. C (CO_3_^2-^) denoted impurities (e.g., MgCO_3_) from MPO hydrogel samples. C_Me_ denoted methanol (residual solvent) from MPO hydrogel samples. (d) Selected regions of the ^13^C CP-TOSS NMR spectra highlighting the alteration of peaks. (e) Raman spectra of MPO hydrogel and ARG, and the MPO NP for comparison. (f) FT-IR spectra of MPO hydrogel and ARG, and the MPO NPs for comparison.

Another key component contributing to the hydrogelation process was the arginine ligand. Arginine possesses a molecular structure with the guanidino and amino/carboxyl terminal groups, which enable it to interact with various residue groups [32]. Notably, the positively charged guanidino group, due to its planar structure formed by the three nitrogen atoms, can readily form strong hydrogen bonds with the negatively charged peroxide groups (-O_2_) (Fig. S14a–b). Furthermore, the amino and carboxyl groups at the terminals are capable of coordinating with the positively charged magnesium ions (Mg^2+^), leading to stable ionic interactions [33]. Based on this, arginine is envisioned as a linker that facilitate the crosslinking of molecular MPO and expand the hydrogel network. As illustrated in Fig. 2a, molecular MPO is represented as a chain-like structure with repeating [-Mg-O_2_-] units to visualize the gel formation process (although the actual molecular conformation of MPO may differ, this representation aids in understanding the crosslinking mechanism). Initially, the guanidino terminal of arginine formed robust hydrogen bonds with the peroxide groups of (MgO_2_)_n_. Subsequently, the amino or carboxyl terminal of another arginine molecule coordinated with neighboring metal ions (Mg), stabilizing the spatial structure of the [-Mg-O_2_-] unit. As more arginine molecules interacted with the (MgO_2_)_n_ chains via hydrogen bonding and ionic coordination, spatial stacking interactions may also occur, ultimately leading to the formation of a three-dimensional crosslinked network (Fig. 2b, Fig. S14c–d).

Solid-state NMR (ssNMR) spectroscopy was employed to investigate the specific interactions between arginine (ARG) and MPO species within the hydrogels, with a focus on hydrogen bonding and ionic coordination. The ^13^C cross-polarization with total suppression of sidebands (CP-TOSS) NMR spectrum of the MPO hydrogel (Fig. 2c) largely resembled that of the pure ARG linker. Notably, the resonances in the hydrogel spectrum exhibited significant line broadening compared to the sharp peaks of crystalline ARG, consistent with the formation of an amorphous cross-linked network. Specifically, the distinct signals for the β-carbon (C_β_, site 3) and γ-carbon (C_γ_, site 4) coalesced into a single broad peak, indicating a convergence of their chemical environments due to extensive intermolecular interactions upon gelation. Critically, a significant downfield shift of ∼3.2 ppm was observed for the carbonyl carbon (site 1), from 179.8 ppm in pure ARG to 183.0 ppm in the hydrogel (Fig. 2d). This pronounced deshielding was consistent with a reduction in electron density at the carbonyl group, unequivocally supporting ionic coordination between the carboxylate oxygen and the magnesium ion of the MPO species. Furthermore, the guanidino carbon (C_ζ_, site 6) also exhibited a downfield shift, suggesting a decreased electron density at this site due to the involvement of the guanidino group in hydrogen bonding with MPO. Minor signals at 170 ppm and 78 ppm were assigned to trace impurities of magnesium carbonate and residual methanol, respectively. The formation of these interactions was further corroborated by ^1^H ssNMR spectra (Fig. S15).

Raman and FT-IR spectroscopy provided complementary evidence for the MPO-ARG interactions. Raman spectra showed significant shifts and broadening of key vibrational modes (Fig. 2e). The O-O stretching vibration of the peroxo group shifted from 868 cm^−1^ in MPO NP to 875 cm^−1^ in the hydrogel, indicating its role as a hydrogen bond acceptor, likely with the N-H donors of the arginine guanidinium group. Concurrently, the sharp N-H stretching modes of pure arginine (3290 and 3347 cm^−1^) were replaced by a broadened band centered at 3405 cm^−1^, characteristic of strongly hydrogen-bonded N-H donors. Additional evidence came from the O-H region, where a sharp peak at 3649 cm^−1^ broadened and shifted to 3634 cm^−1^, indicating incorporation into the gel's hydrogen-bonded network. The coalescence of arginine's sharp C-H stretching vibrations into a broad feature at 2931 cm^−1^ further supported a restricted molecular environment consistent with gel formation. Meanwhile, FT-IR analysis showed that the N-H/O-H stretching region exhibited a markedly broadened band at 3442 cm^−1^, indicative of an extensive hydrogen-bonding network (Fig. 2f). Furthermore, compared to pure arginine, the antisymmetric carboxylate stretching vibration exhibited a distinct blue shift and significant broadening at 1636 cm^−1^, providing direct evidence of ionic coordination between the carboxylate group and Mg^2+^, attributable to altered electron density and symmetry upon metal complexation. Together, these spectroscopic data provided consistent and multi-faceted evidence for the dual interaction mechanism underpinning the hydrogel formation.

It is important to note that, unlike conventional hydrogel formation process, the formation of MPO hydrogels involved not only physical crosslinking, but also the chemical generation of the crosslinking agent MPO itself. A key factor ensuring the hydrogelation process is the unique structural form of MPO. Direct complexation of pre-synthesized MPO nanoparticles with arginine could not result in effective hydrogel formation (Fig. S16). During the synthesis of MPO hydrogels, in addition to acting as a crosslinker, arginine further played a critical role as a modifier that restrict the crystallization and growth of MPO. This modification is essential for the formation of the desired MPO structure. Synthesis without arginine (MPO-5) resulted in amorphous MPO aggregates. Besides, the addition of arginine after the reaction was unable to guide the hydrogelation. (Fig. S17).

### 2.3 Decomposition and oxidant neutralization of MPO hydrogels

**Fig. 3:**
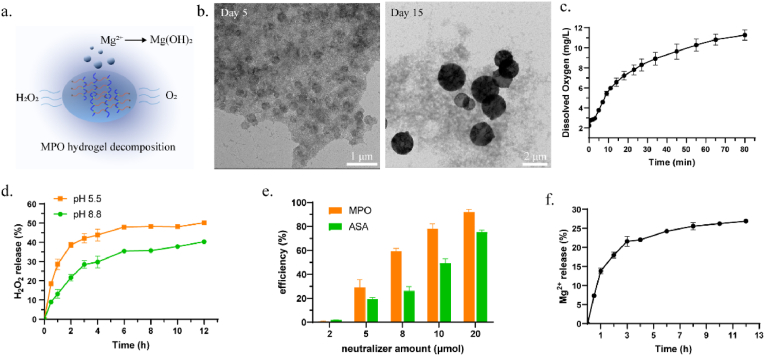
Decomposition and oxidant neutralization study of MPO hydrogel. (a) Schematic illustration of MPO hydrogel decomposition. (b) TEM images of MPO hydrogel at day 5 and day 15. (c) Oxygen release pattern of MPO hydrogel. (d) H_2_O_2_ release of MPO hydrogel at different pH buffers. (e) Neutralizing efficiency of MPO hydrogel or ascorbic acid (ASA) towards NaClO oxidants. (f) Mg^2+^ release of MPO hydrogel.

While the guanidino groups can form robust hydrogen bonds with the peroxide groups of (MgO_2_)_n_, the triple-nitrogen structure of the guanidino group may also promote its decomposition. Compared to the bidentate nitrogen structure of urea, the three nitrogen atoms in the guanidino group exhibit a strong electron-donating effect through resonance, making them more susceptible to protonation. This proton transfer can facilitate the cleavage of the Mg-(O_2_) bond and promote the decomposition of MPO species into H_2_O_2_, water and oxygen. Thus, the hydrogel became less stable when stored at room temperature, and tended to subsequently transfer to Mg(OH)_2_ (Fig. 3a, Fig. S18). Besides, upon supersaturated hydration, the gradual decomposition of MPO hydrogel could be triggered as well, primarily due to the penetrating water molecules accelerating the hydrolytic breakdown of the metastable MPO species (Fig. S19). As shown in Fig. 3b, TEM images revealed the formation of 200 nm disc-like structures on day 5 (Fig. S20). By day 15, larger two-dimensional nanodiscs (1–2 μm diameter) were observed. The XRD pattern confirmed the formation of Mg(OH)_2_ (Fig. S21).

The oxygen release from MPO hydrogels was monitored using a portable dissolved oxygen meter (Fig. 3c). During the first 15 min, the dissolved oxygen concentration rapidly increased to 6.5 mg/L, followed by a slower rise to 11 mg/L, indicating the efficient oxygen supply. The release of H_2_O_2_ was further quantified in different pH buffer solutions (Fig. 3d). Initially, H_2_O_2_ was quickly released during the first 2 h, and then reached a plateau at 6 h. After 12 h, approximately 50 % of H_2_O_2_ was released at pH 5.5, while 40 % was released at pH 8.8. In alkaline conditions, the release of H_2_O_2_ was relatively lower, likely due to its conversion into oxygen. In contrast, at acidic pH, H_2_O_2_ release was nearly doubled at first 2 h, consistent with the tendency of MPO to decompose more readily under acidic conditions. The H_2_O_2_ release profile was further evaluated in simulated physiological media containing serum or lysozyme, revealing a more sustained release behavior (Fig. S22). This is likely attributed to protein adsorption onto the hydrogel surface, which moderates surface erosion and H_2_O_2_ diffusion. Besides, the diluted MPO solution was found to release H_2_O_2_ more rapidly than intact MPO hydrogel, as the supramolecular network physically restricts water accessibility to the MPO active sites (Fig. S23). The release of Mg^2+^ was also monitored over time (Fig. 3f). Similar to the H_2_O_2_ release pattern, Mg^2+^ release was rapid in the first 3 h, followed by a slower release phase. By the 12-h mark, the cumulative release of Mg^2+^ increased to 26 %. The amount of Mg^2+^ released was lower than that of H_2_O_2_, which may be attributed to the *in situ* formation of Mg(OH)_2_ nanodiscs, thereby limiting the release of free Mg^2+^.

While H_2_O_2_ can act as a reducing agent to react with oxidants and form biocompatible water and oxygen, MPO hydrogels are expected to serve as potential chemical neutralizers, efficiently scavenging reactive oxidants such as sodium hypochlorite (NaClO). To evaluate this capability, NaClO was used as a model oxidant, with ascorbic acid (ASA) serving as a comparative control (Fig. 3e). The results demonstrated that the MPO hydrogel exhibited significantly higher oxidant-scavenging efficiency than ASA. This superior performance may be attributed to the clean and efficient redox reaction that neutralizes NaClO without generating persistent oxidative byproducts (Eq. (1)). In contrast, ASA likely induced side reactions, regenerating other oxidative species (e.g., Cl_2_), which remaining as oxidative substances and result in a lower apparent scavenging efficiency. These results suggested that MPO hydrogels hold promise as a first-aid treatment for oxidant-induced chemical burns.

(Eq. 1) NaClO + H_2_O_2_ → NaCl + H_2_O + O_2_

### 2.4 *In vitro* study of oxidant neutralization and wound healing promotion

**Fig. 4:**
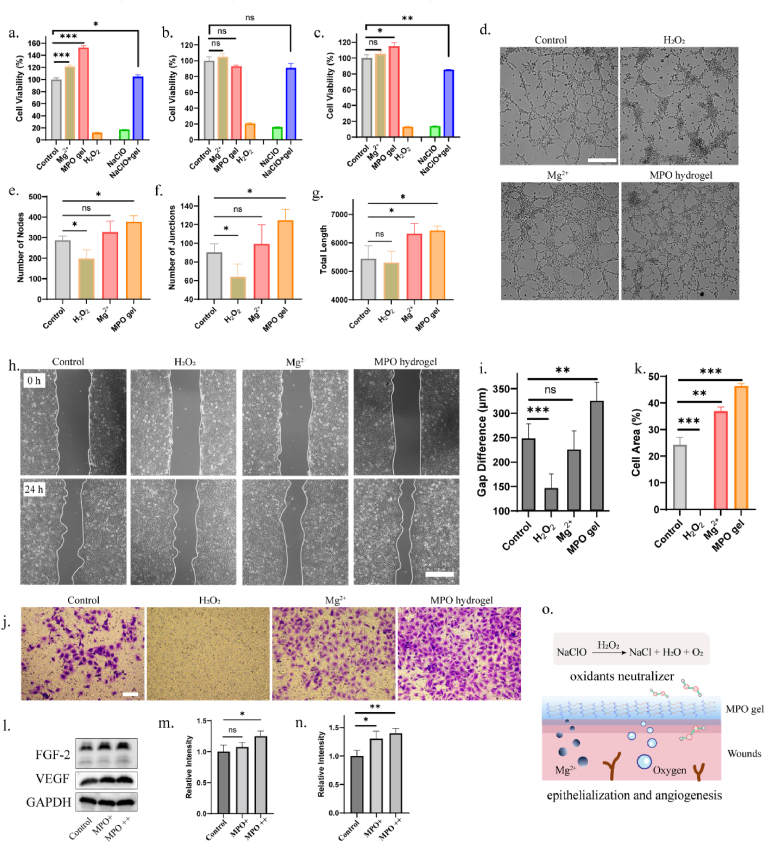
The oxidant neutralization and wound healing promotion capabilities of MPO hydrogel. (a–c) CCK8 assays of HACAT (a), HEUVC (b), and HDF (c) cells with the treatment of different agents (n = 6). ∗p < 0.05, ∗∗p < 0.01, ∗∗∗p < 0.001, by one-way ANOVA tests. (d) Images of tube formation assay after treated with varied agents. scale bar: 500 μm. (e–g) Quantification analysis of the node numbers (e), junctions (f) and total length (g) in the tube formation images (n = 4). ∗p < 0.05, ∗∗p < 0.01, ∗∗∗p < 0.001, by one-way ANOVA tests. (h) Images of wound scratch assay after treated with varied agents. scale bar: 500 μm. (i) Quantification analysis of the gap difference in different groups (n = 4). ∗p < 0.05, ∗∗p < 0.01, ∗∗∗p < 0.001, by one-way ANOVA tests. (j) Images of transwell migration assay after treated with varied agents. scale bar: 100 μm. (k) Quantification analysis of the cell area in different groups (n = 3). ∗p < 0.05, ∗∗p < 0.01, ∗∗∗p < 0.001, by one-way ANOVA tests. (l) Western blotting in HEUVC cells after treated with different amount of hydrogel. (m–n) Quantification of the relative expression of VEGF (m), FGF-2 (n) proteins. (o) Schematic illustration of MPO hydrogel neutralizing oxidant and promoting wound healing.

Since oxygen and Mg^2+^ have been widely reported to promote epithelialization and angiogenesis as essential processes for effective wound healing, MPO hydrogel were supposed as a potential agent for wound healing advance. To evaluate its effects, *in vitro* assays were performed. When co-culturing MPO hydrogel with cells for 24 h, CCK-8 assays showed a noticeable proliferative effect on HACAT cells, and negligible changes observed in HEUVC and HDF cells, indicating the excellent biocompatibility of MPO hydrogel (Fig. 4a–c). In contrast, co-incubation with H_2_O_2_ induced cell death in all three cell types, further demonstrating that the controlled release property of MPO hydrogel secured the cell survival. Moreover, MPO hydrogel exhibited remarkable oxidant neutralization capabilities. MPO hydrogel itself and the released H_2_O_2_ act as chemical neutralizers, scavenging highly reactive oxidants while decomposing into water and oxygen. For instance, co-culturing sodium hypochlorite with cells leads to cell death. However, the addition of MPO hydrogel significantly enhanced cell survival (Fig. 4a–c).

To assess the angiogenic potential of the MPO hydrogel, a tube formation assay was conducted. As illustrated in Fig. 4d, the Mg^2+^ group and the MPO group demonstrated distinct effects on endothelial cell tubulogenesis, as evidenced by the formation of more intricate and interconnected network structures compared to the control group (Fig. S24). Quantitative analysis of nodes, junctions and total length reflected the network complexity, connectivity, and spatial coverage, respectively, with the MPO group showing the greatest improvement (Fig. 4e–g). In contrast, the H_2_O_2_ group formed isolated clusters, likely due to oxidative stress impairing cell function and matrix remodeling. Additionally, the wound scratch assay revealed the smallest gaps after 24 h of incubation with the MPO hydrogel, with a 20 % greater reduction in gap size compared to the control group, indicating the enhanced cell migration (Fig. 4h–i, Figure S25). While the H_2_O_2_ group exhibited minimal closure and the Mg^2+^ group showed insignificant improvement over the control, the MPO hydrogel's dual release of oxygen and Mg^2+^ synergistically promoted migration, highlighting its potential for accelerating wound healing and vascularization. To assess the migratory potential more precisely, a transwell migration assay was performed. The results showed that the MPO hydrogel treatment significantly enhanced cell migration, with the number of cells on the lower side of the membrane nearly doubling compared to the control (Fig. 4j and k). The expression of angiogenic growth factors were further elucidated. Treatment with MPO hydrogel dose-dependently increased the expression of vascular endothelial growth factor (VEGF) and basic fibroblast growth factor (FGF-2), key mediators of reepithelialization and angiogenesis during wound healing (Fig. 4l–n). The upregulation of VEGF and FGF-2 in response to the released oxygen and Mg^2+^ may be attributed to increased phosphorylation of Erk1/2 (p-Erk1/2) (Fig. S26). Previous studies have demonstrated that the activation of Erk1/2 signaling pathway upregulates the expression of angiogenic growth factors in cells [34,35]. Besides, the unchanged expression of heme oxygenase-1 (HO-1) following MPO hydrogel treatment suggested that the hydrogel would not induce cellular oxidative stress or inflammation (Fig. S26). All the results suggested the great potential of MPO hydrogel for wound healing advance, in which the decomposition products Mg^2+^ and oxygen accelerated the local angiogenesis and further contributed to epithelialization, and H_2_O_2_ generation would potentially become chemical neutralizer to scavenge extra reactive oxidants (Fig. 4o).

### 2.5 Mechanism study by RNA sequencing

**Fig. 5:**
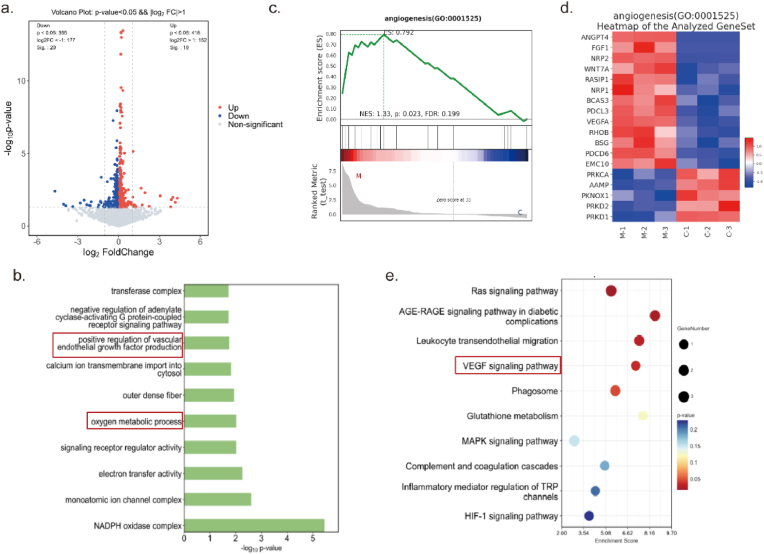
Mechanism study based on RNA sequencing (n = 3). (a) Volcano plot of upregulated (red) and downregulated (blue) genes. (b) GO enrichment analysis of DEGs (fold changes ≥1.5). (c) GSEA analysis of the genes related to angiogenesis. (d) Heatmap of the analyzed geneset of angiogenesis. (e) Kyoto Encyclopedia of Genes and Genomes (KEGG) analysis of DEGs.

To elucidate the mechanisms by which MPO hydrogel promoted angiogenesis and epithelialization, RNA sequencing (RNA-seq) was conducted comparing the control and MPO-treated groups. In total, 416 genes were up-regulated and 355 were down-regulated in the MPO group relative to the control (Fig. 5a). GO enrichment analysis indicated that the differentially expressed genes (DEGs) were associated with biological processes including positive regulation of vascular endothelial growth factor (VEGF) production and oxygen metabolic process, as well as molecular functions such as electron transfer activity and components of the NADPH oxidase complex (Fig. 5b). Gene Set Enrichment Analysis (GSEA) further revealed significant enrichment of the gene set “oxygen metabolic process” and enrichment for “positive regulation of vascular endothelial growth factor production” in the MPO group (Fig. 5c, Fig. S27). These findings implied a coordinated upregulation of pro-angiogenic signaling pathways. A heatmap analysis of angiogenesis-related genes demonstrated elevated mRNA expression of pro-angiogenic factors in the MPO group, including VEGFA, ANGPT4, FGF1, NRP1, NRP2, WNT7A, RHOA and BSG, while negative regulators such as PRKCA, PRKD1 and PRKD2 were downregulated (Fig. 5d, Fig. S27). KEGG pathway enrichment analysis highlighted the VEGF signaling pathway as a key pathway modulated by MPO treatment (Fig. 5e). These transcriptomic changes were consistent with elevated protein levels of VEGF and FGF2 (Fig. 4l), supporting enhanced angiogenic potential. Collectively, these results suggest that MPO hydrogel facilitated wound healing by enhancing genes related to angiogenesis and epithelialization.

### 2.6 MPO hydrogel for chemical burn treatment *in vivo*

The growth of the chemical industry and the increasing use of chemicals in warfare have made chemical burns a common occupational risk. Workers in fields such as chemical manufacturing, laboratory work, and firefighting are especially prone to these injuries due to exposure to hazardous substances. Accidental exposure to chemicals can lead to severe skin burns. Emergency treatment for chemical burns typically involves extensive rinsing with water, followed by the application of neutralizing agents. However, chemicals that have penetrated the skin may not be fully removed by washing alone, leading to the potential for secondary chemical burns. Furthermore, the development of effective neutralizing agents remains limited, particularly for handling risk of highly oxidative chemical solutions. For example, sodium hypochlorite, a widely used disinfectant, can cause severe oxidative skin burns if misused [36]. In light of this, we have selected sodium hypochlorite as a model compound to evaluate the potential of MPO hydrogel as a neutralizer to mitigate chemical burns and promote wound healing.

The *in vivo* biosafety of the MPO hydrogel was first evaluated. Compared to the control group, topical administration of the hydrogel did not induce any short- or long-term systemic toxicity. Hematological and serum biochemical analyses revealed values within normal reference ranges (Figs. S28–S29). Mouse body weight remained stable throughout the evaluation period, and histological examination (H&E staining) of major organs showed no abnormalities (Fig. S30a and S30d). Furthermore, H&E staining of skin tissue at the site of topical treatment revealed no signs of inflammatory infiltration (Fig. S30e). Serum levels of inflammatory cytokines (TNF-α and IFN-γ) were comparable to those in the control group (Fig. S30b and S30c), indicating that MPO hydrogel treatment did not provoke any local or systemic inflammatory responses.

**Fig. 6:**
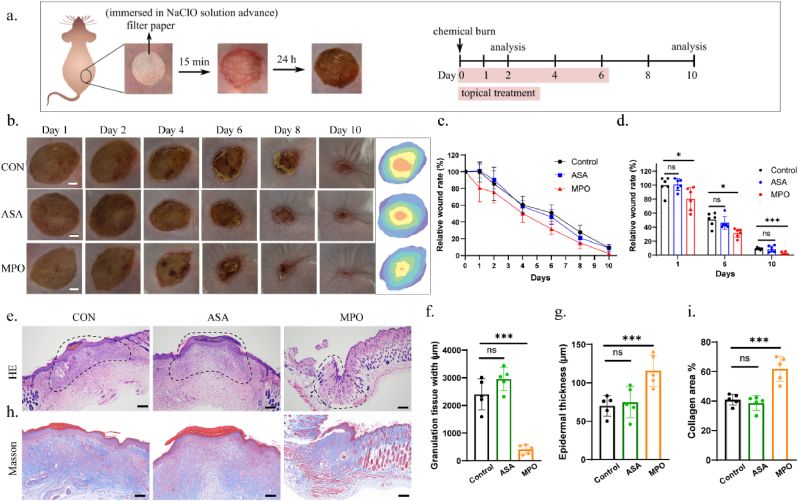
MPO hydrogel for chemical burn treatment *in vivo*. (a) Illustration of establishing the chemical burn model in the mouse skin and the following treatment procedure. (b) Wound images after different treatment on chemical burn skin wound repair. Scale bar: 2 mm. (c) Wound closure rate after different treatment on chemical burn skin wound (n = 6). (d) Relative wound area for different groups at day 1, 6 and 10 (n = 6). ∗p < 0.05, ∗∗p < 0.01, ∗∗∗p < 0.001, by one-way ANOVA tests. (e) H&E staining of the skin wound tissues (circled area) on day 10 after different treatment. Scale bar: 200 μm. (f) Quantification analysis of the granulation tissue width of H&E staining (n = 5). ∗p < 0.05, ∗∗p < 0.01, ∗∗∗p < 0.001, by one-way ANOVA tests. (g) Quantification analysis of the epidermal thickness of H&E staining (n = 5). ∗p < 0.05, ∗∗p < 0.01, ∗∗∗p < 0.001, by one-way ANOVA tests. (h) Masson staining of the skin wound tissues on day 10 after different treatment. Scale bar: 200 μm. (i) Quantification analysis of the collagen area of Masson staining (n = 5). ∗p < 0.05, ∗∗p < 0.01, ∗∗∗p < 0.001, by one-way ANOVA tests.

A chemical burn wound model was established by directly applying 10 % sodium hypochlorite to the shaved dorsal skin of mice (Fig. 6a). MPO hydrogel was then applied to cover the wound area, with fresh hydrogel being replaced every two days (Fig. S31). As a comparison control, ascorbic acid (ASA), known for its well-established antioxidant properties, was chosen for comparison. Wound healing was monitored over a ten-day observation period (Fig. 6b). During the period, the body weight of all three groups remained unchanged, indicating good biocompatibility of the applied agents (Fig. S32). On the first day following treatment, the wound area in the MPO group was significantly smaller than in the other two groups, while the ASA group showed similar size to the control group (Fig. 6d). This suggested that appropriate neutralizing agents can greatly reduce skin damage in the early stages of oxidative injury. Two days after treatment, all mice began to form scabs, but the scabs in the MPO group were noticeably thinner than in the other groups. By day ten, the wound in the MPO group was almost completely healed, whereas the wounds in the other two groups had not fully healed (Fig. 6c).

Skin tissues from the wound areas of each group were then collected at day ten for hematoxylin and eosin (H&E) staining and Masson's trichrome staining to assess wound healing. H&E staining revealed that the wounds in the MPO group had completely healed, with well-regenerated epidermis and visible regeneration of skin appendages such as hair follicles (Fig. 6e, Fig. S33). The epidermal thickness had returned to normal range, and the dermal structure was orderly with minimal infiltration of inflammatory cells (Fig. 6g). In contrast, wounds in the other two groups still exhibited prominent scabs, thinner epidermal layers, and areas filled with a large number of cells (e.g. inflammatory or fibroblast cells), with granulation tissue width approximately five times that of the MPO group (Fig. 6f), indicating that the wounds were still in the process of healing and had not yet matured. Masson staining further revealed that the wound area in the MPO group was densely populated with well-organized collagen fibers, indicative of a mature and efficient healing process (Fig. 6h, Fig. S34). In contrast, the wounds in the other two groups showed limited collagen deposition with a more disorganized arrangement, suggesting slower healing and incomplete tissue repair (Fig. 6i).

### 2.7 Histological immunological staining analysis

**Fig. 7:**
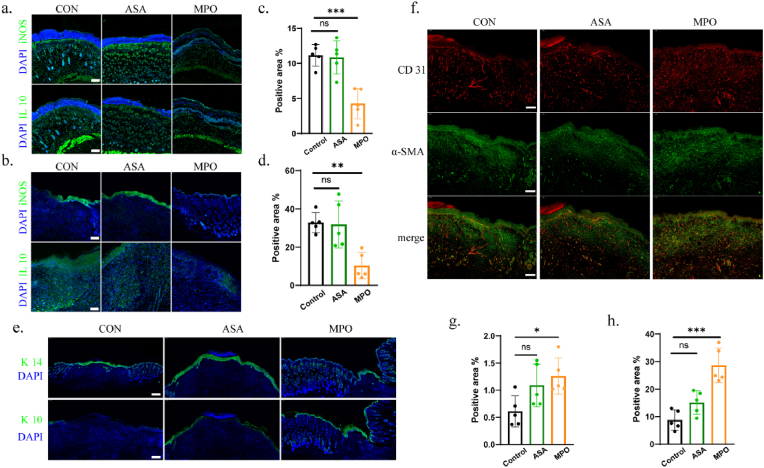
Immunofluorescence staining analysis of the wounds after different treatment. (a) IF staining of iNOS and IL-10 at day 2. Scale bar: 200 μm. (b) IF staining of iNOS and IL-10 at day 10. Scale bar: 200 μm. (c) Quantification analysis of iNOS at day 10 (n = 5). ∗p < 0.05, ∗∗p < 0.01, ∗∗∗p < 0.001, by one-way ANOVA tests. (d) Quantification analysis of IL-10 at day 10 (n = 5). ∗p < 0.05, ∗∗p < 0.01, ∗∗∗p < 0.001, by one-way ANOVA tests. (e) IF staining of K14 and K10 at day 10. Scale bar: 500 μm. (f) IF double staining of CD31 and α-SMA at day 10. Scale bar: 200 μm. (g) Quantification analysis of CD31 at day 10 (n = 5). ∗p < 0.05, ∗∗p < 0.01, ∗∗∗p < 0.001, by one-way ANOVA tests. (h) Quantification analysis of α-SMA at day 10 (n = 5). ∗p < 0.05, ∗∗p < 0.01, ∗∗∗p < 0.001, by one-way ANOVA tests.

In an oxidant-induced chemical burn, rather than the full-thickness injury, the damage induced by oxidant (NaClO) can be progressive and depth-dependent. At the initial stage, the injury could be seen primarily epidermal, characterized by necrosis of the epidermis and the formation of a superficial eschar. However, as the residual oxidant continue to penetrate deeper tissue layers over time, damages could progressively extend to the dermis and even underlying structures through sustained oxidative stress. On day 2, IF staining of tissues revealed a significant increase in the expression of the pro-inflammatory factor iNOS in the dermis of both the control and ASA groups, indicating severe oxidative injury induced by the oxidants (Fig. 7a). Magnified images facilitated clearer view that the inflammation was presented in follicular tissues, suggesting that oxidants may penetrate through the hair follicles (Fig. S35). Additionally, a dense blue signal zone appeared on the tissue surface, representing scab formation due to the accumulation of necrotic cells. Concurrently, IL-10 expression was also increased, suggesting an attempt to suppress excessive inflammatory responses. In contrast, the MPO group exhibited a marked reduction in iNOS expression and a noticeable decrease in necrotic tissue on the surface, indicating superior neutralization functions towards oxidative injury. On day 10, iNOS expression was primarily concentrated in the epidermis of the control and ASA groups, while IL-10 expression was widely distributed across the tissue, suggesting that the wound was still in an incomplete healing state with ongoing tissue repair (Fig. 7b, Fig. S36). In comparison, both iNOS and IL-10 expression were significantly reduced in the MPO group on day 10, reflecting that the tissue had undergone complete healing (Fig. 7c and d). Moreover, the MPO group demonstrated higher expression of the epithelial marker K10 and reduced expression of K14, suggesting that the treatment effectively promoted skin epithelialization (Fig. 7e). In contrast, the control group showed elevated K14 expression and lower K10 expression, indicating that healing was still incomplete and the epidermal barrier function was under restoration. Additionally, CD31 expression was slightly elevated in the MPO group, and α-SMA expression was significantly upregulated, indicating that MPO treatment enhanced the activity of myofibroblasts, accelerated neovascularization, and promoted tissue remodeling (Fig. 7f–h, Fig. S37). These results collectively suggest that MPO hydrogel effectively neutralized oxidative injury and accelerated wound repair.

## 3 Discussion

The developed MPO supramolecular hydrogel was primarily formed through physical crosslinking via hydrogen bonding and ionic coordination, rather than covalent bonds, resulting in a soft, easily deformable structure with low mechanical strength. Similar to gelatin-based hydrogels, this inherent softness limited conventional macromechanical characterization, but endowed the material with notable shear-thinning and thixotropic behavior (Fig. 1i, Fig. S7), allowing reversible gel-sol transitions under shear stress and rapid self-recovery upon resting. Such dynamic responsiveness made it highly suitable for biomedical applications such as wound dressings or drug delivery systems. Furthermore, the hydrogel underwent spontaneous decomposition into H_2_O_2_, Mg^2+^, oxygen, and water, a process accompanied with saturated swelling and mediated by the disruption of intermolecular interactions within the gel network. This degradability was functionally advantageous. For example, under physiological conditions, the released Mg^2+^ and oxygen promoted wound healing, while in NaClO-induced chemical burns, the released H_2_O_2_ directly and rapidly neutralized residual oxidants via a reaction yielding only non-oxidative products. This multifaceted functionality highlights the potential of MgO_2_ hydrogels as first-aid treatments for oxidant-induced chemical injuries.

Compared to conventional peroxide-based materials that release H_2_O_2_ typically relying on passive diffusion or bulk erosion of pre-synthesized peroxides, the MPO hydrogel exhibited a fundamentally different mechanism. Its H_2_O_2_ release was governed by the dynamic recrystallization of metastable MPO species within the supramolecular network, a process finely modulated by physiological cues such as moisture, pH, and local redox. Besides, unlike conventional systems that passively encapsulate pre-formed MgO_2_ nanoparticles within inert hydrogel carriers (e.g., alginate or chitosan), which often leading to burst release and crosslinker-related biocompatibility concerns, this hydrogel system utilized metastable MgO_2_ species as dynamic structural nodes in a crosslinker-free, self-assembled network. This design eliminated the need for extrinsic carriers, achieving enhanced functional integration and improved release kinetics unattainable with conventional nanoparticle-loaded hydrogels.

Building upon the unique features of the MPO hydrogel, its performance in a physiologically relevant animal model of NaClO-induced chemical burn was evaluated. This model is characterized by progressive, depth-dependent tissue damage rather than immediate full-thickness injury. Initially, the damage is primarily epidermal, presenting as necrosis and superficial eschar formation. Then, residual NaClO continues to penetrate deeper tissues over time, inducing sustained oxidative stress that leads to progressive dermal and subdermal damage. This pathogenesis explains why, in the early stages, immunofluorescence staining revealed intense inflammatory activity and oxidative stress in the dermis rather than overt structural destruction (Fig. 7a), and then the injury progressed to exhibit characteristics of a full-thickness skin loss model, as confirmed by H&E staining (Fig. 6e).

Given the persistent oxidative challenge posed by penetrating residual oxidants, an ideal therapeutic agent must not only neutralize surface contaminants but also penetrate tissue layers to eliminate deeply infiltrated oxidants, and yielding biocompatible byproducts. Conventional irrigation solutions like bicarbonate are ineffective against hypochlorite, and common burn dressings such as silver-based dressings or hyaluronic acid gels, though beneficial for infection control or hydration, lack oxidant-neutralizing capability. This aligns with the animal results, which showed no significant improvement in wound closure with these conventional treatments compared to untreated controls (Fig. S38 and S39). In this study, ASA was selected as a comparative control due to its recognized antioxidant properties and theoretical potential to neutralize NaClO. However, ASA failed to elicit the anticipated therapeutic effect, likely owing to its slower reaction kinetics and the generation of persistent chlorinated oxidative byproducts, which ultimately prolong oxidative stress.

In contrast, the MPO hydrogel enabled sustained, localized release of H_2_O_2_, which reacted directly and rapidly with NaClO to produce fully biocompatible products (NaCl, H_2_O, and O_2_). This efficient redox reaction not only neutralized the oxidant but also supplied oxygen conducive to healing. *In vitro* studies confirmed the superior neutralizing efficiency of MPO over ASA at equivalent doses (Fig. 3e), underscoring the advantages of targeted, reaction-efficient design in chemical burn management. Besides, it is important to address the potential cytotoxicity of H_2_O_2_. While concerns exist in conventional wound contexts, the risk-benefit profile shifts fundamentally in chemical burn scenarios characterized by ongoing oxidant penetration. Here, MPO-derived H_2_O_2_ served a critical neutralizing function, rapidly converting residual NaClO into harmless compounds and preventing deep tissue damage. Furthermore, the treatment protocol was designed to limit hydrogel application after the acute phase, minimizing prolonged H_2_O_2_ exposure once oxidants are cleared. Histopathological and cytokine (TNF-α) analyses confirmed the biosafety of the MPO hydrogel (Fig. S29), showing no abnormal inflammation or elevated pro-inflammatory markers compared to untreated tissues.

Aside from the promising biosafety and efficacy profiles of the MPO hydrogel, certain limitations must be acknowledged to guide future development. A primary challenge lied in the inherent instability of MPO species under physiological conditions, where factors such as ions, enzymes, and acidic microenvironments could accelerate its decomposition. Although low-temperature storage (e.g., at 4 °C) was shown to preserve over 40 % of MgO_2_ purity after 10 days (Fig. S18), which provide a practical near-term solution, long-term stabilization strategies remain essential. Future efforts may include formulation optimization and lyophilization to enhance shelf stability. Moreover, to improve functional stability under application conditions, the introduction of protective buffer layers represents a promising approach. For instance, encapsulating MPO microgels within an alginate-based hydrogel to form core–shell microspheres could shield the active material and ensure more consistent release. Beyond stability enhancement, future iterations could significantly expand functionality through integrated controlled-release mechanisms (e.g., light-, pH-, or enzyme-triggered systems) and auxiliary bioactive agents (e.g., antimicrobial peptides or growth factors). The existing supramolecular architecture offers a highly modular platform amenable to such innovations. Incorporating oxidative stress-sensitive linkers or photoactivatable components could enable precise spatiotemporal control over H_2_O_2_ and O_2_ release, paving the way for adaptive, multifunctional wound management systems that respond dynamically to the wound microenvironment.

## 4 Conclusion

This study reports a kind of MgO_2_-assembled supramolecular hydrogel, and further explores its therapeutic potential for chemical burn management. The hydrogel network was constructed through non-covalent interactions, including hydrogen bonding and Mg^2+^-mediated coordination between MPO and linker molecules. Notably, the synthesis enabled the *in-situ* formation of MPO species, which served as critical network nodes to drive the self-assembly process. Besides, it was found that the choice of linker molecules significantly influenced the mechanical strength of the resulting hydrogel. The hydrogel exhibited dynamic stability at ambient conditions, with MPO nodes undergoing controlled recrystallization to release oxygen, H_2_O_2_ and Mg^2+^. This characteristic endowed the hydrogel with the ability to neutralize oxidants (e.g., NaClO) through redox reactions with hydrogen peroxide while simultaneously generating oxygen. The oxygen and Mg^2+^ supplying jointly fostered a pro-healing microenvironment by enhancing tissue regeneration, making the hydrogel a promising candidate for emergency care towards chemical burns.

In a NaClO-induced chemical burn mouse model, the hydrogel demonstrated superior *in vivo* performance, achieving rapid oxidant neutralization and accelerated chemical burn wound closure. To our knowledge, this work represents the first successful fabrication of a MgO_2_-integrated supramolecular hydrogel without chemical crosslinkers. The unique combination of self-assembly-driven fabrication, on-demand oxygen release, and therapeutic functionality opens new avenues for advancing oxygen-supplying hydrogels in trauma care. This platform not only enriches the theoretical framework of dynamic supramolecular materials but also holds translational promise for developing next-generation first-aid formulations targeting chemical injuries.

## 5 Materials and methods

### 5.1 Materials

Magnesium chloride hexahydrate, magnesium sulfate anhydrous, sodium citrate dihydrate, ascorbic acid, sodium bicarbonate, hydrogen peroxide, ammonia solution (≥28 %) were obtained from General-reagent. Methanol (99.9 %), sodium citrate, L-arginine, citrulline, argininamide and arginine methyl ester dihydrochloride, sodium alginate and alizarin red were purchased from Macklin. Sodium hypochlorite pentahydrate, sodium hyaluronate, lysozyme, Tris-HCl buffer (pH 5.5), cerium sulfate standard solution (0.1000 mol/L) was purchased from Aladdin. Ag-based dressing was obtained from Innomed. HACAT, HUVEC, HDF cells were obtained from Cell Bank, Chinese Academy of Sciences. Cell Counting Kit-8 (CCK-8) was obtained from Beyotime Biotechnology. Matrigel was purchased from Corning. Antibody: p44/42 MAPK (Erk1/2) (#4695), Phospho-p44/42 MAPK (Erk1/2) (#4370) were obtained from CST. VEGF (ab150375) was obtained from Abcam. FGF-2 (ET1703-18), HO-1 (HA721854) were obtained from HUABIO. iNOS (GB11119), IL-10 (GB11108), K10 (GB112105), K14 (GB11803), CD31 (GB15063), α-SMA (GB111364) were obtained from ServiceBio. Mouse TNF-α and IFN-γ ELISA kit was purchased from Yeasen.

### 5.2 Synthesis of MPO hydrogels

85 mg L-arginine and 0.8 mL of ammonia solution were dissolved in 15 mL of methanol in a round-bottom flask. Subsequently, 0.1 mL of hydrogen peroxide was added and mixed thoroughly. The mixture was then placed in an ice-water bath. Meanwhile, a 55 mM magnesium chloride aqueous solution was prepared separately. Under gentle stirring, the magnesium chloride solution was added dropwise to the flask using an injection pump at a rate of 0.4 mL/min, with the addition completed over 23 min. Afterwards, the reaction mixture was allowed to react in the ice-water bath for 24 h. The product was ultracentrifuged for 15 min and washed with deionized water, and finally redispersed in 3 mL of deionized water to undergo the hydrogelation. The final product was stored at 4 °C for further purpose.

To investigate the role of arginine, varying amounts of arginine (145 mg, 85 mg, 55 mg, 36 mg, 24 mg, 0 mg) were added, with the products denoted as MPO-0, MPO-1, MPO-2, MPO-3 MPO-4 and MPO-5, respectively. Additionally, arginine was substituted with either citrulline, argininamide or arginine methyl ester, maintaining the same molar concentrations for comparison.

The concentration of the hydrogel was determined based on its hydrogen peroxide content, as previously reported, using the cerium sulfate method [30]. Briefly, in the acidic environment, hydrogen peroxide stoichiometrically reduces cerium (IV) ions to cerium (III) ions. Within a specific concentration range, the UV absorbance of cerium (IV) ions at 350 nm is linearly correlated with their concentration. Using a microplate reader, the amount of cerium (IV) ions consumed can be quantified, allowing the hydrogen peroxide content in the sample to be calculated.

### 5.3 Synthesis of MPO nanoparticles

The pre-synthesized MPO nanoparticles were prepared as following: 5 mmol of magnesium sulfate solution was mixed with 5 wt% sodium citrate. After stirring evenly, ammonium was added to the mixture. The suspension was stirred for 20 min, followed by the addition of 4 mL H_2_O_2_. The system was allowed to react for 1 h. Finally, the resulting precipitate was centrifuged and washed several times to obtain pre-synthesized MPO nanoparticles.

### 5.4 Characterizations

The micro-morphology and elemental analyses of MPO gels were conducted using a field-emission transmission electron microscope (TALOS F200X) equipped with an energy-dispersive X-ray spectrometer. Typically, the hydrogel was placed in a dish, and a copper grid was gently contacted with the hydrogel surface using fine forceps to collect a thin sample film. Excess water was then removed by infrared lamp irradiation. The dried copper grid was examined using the TEM.

The gels were further freeze-dried for subsequent characterizations. SEM images were acquired on a Scanning Electron Microscope (RISE-MAGNA, TESCAN). XRD pattern was carried out by X-ray diffraction (Mini Flex 600, Rigaku). Raman spectra were acquired on a Renishaw inVia Qontor (UK). Fourier-transform infrared (FTIR) was collected by Nicolet 6700 (THERMO FISHER, USA). X-ray photoelectron spectroscopy (XPS) measurements were recorded with an AXIS UltraDLD (Shimadzu) X-ray photoelectron spectrometer. The thermogravimetric and differential scanning calorimetry analysis was obtained on a STA 449 F3. The ssNMR experiments were executed on a Bruker AVANCE NEO 600WB spectrometer, operating at frequencies of 600.43 MHz for 1H and 150.99 MHz for ^13^C, utilizing a MASDVT600W2 BL3.2X/Y/H probe. The magic angle spinning (MAS) unit was adjusted to rotate at a rate of 15 kHz. Single-pulse sequences were employed to acquire the ^1^H MAS spectra. In acquiring the ^13^C CP-TOSS spectra, a ramped amplitude ^1^H pulse was applied during the contact time (t_c_ = 2000 μs) to mitigate spin modulation under the Hartmann-Hahn condition. Calibration of the chemical shift scale was achieved using adamantane as a reference, with its ^13^C and ^1^H signals set at 38.5 ppm and 1.848 ppm, respectively.

### 5.5 Purity of MPO hydrogels

MPO hydrogel samples were dissolved in sulfuric acid solution (0.5 mol/L), then the hydrogen peroxide content was quantified using the cerium sulfate method, and total Mg content was measured by ICP-OES. The purity of the MPO hydrogel can be calculated as:

Purity (%) = [Peroxide content (mol)] / [Total Mg content (mol)] × 100

where "peroxide" represents the molar amount of hydrogen peroxide in the sample (used as a proxy for MgO_2_), and "Mg" stands for the total molar amount of magnesium ions present (representing both MgO_2_ and magnesium-containing impurities).

### 5.6 Accelerated stability study

Fresh MPO hydrogel was stored at two different temperatures (4 °C and 25 °C). At predetermined time points, the samples were thoroughly mixed, and then 20 mg of each sample was dissolved in 1 mL of dilute sulfuric acid solution. The hydrogen peroxide content and total Mg content were subsequently measured, and the purity of the hydrogel was calculated.

### 5.7 Rheology

Rheological behaviors were investigated using a DHR10 Rotational Rheometer (TA Instrument, USA). The storage and loss modulus versus shear strain were recorded at 25 °C, with a shear strain range from 0.001 % to 100 %. Viscosity and stress as a function of shear rate were recorded at 25 °C, with a shear rate from 0.01 to 100 HZ. Oscillatory recovery test was measured at 25 °C using a ‘peak hold’ procedure, with the shear rate repeatedly switching between 0.01 and 10 Hz.

### 5.8 Injectability evaluation of hydrogels

Colorless MPO gels were stained with alizarin red for subsequent visualization. The gel was then loaded into a 10 mL syringe and extruded through the syringe's nozzle without the use of any additional attachments such as a needle or pillow. Upon extrusion, the shape was observed, and it was noted that the gel lost its fluidity within 1 min after being extruded, indicating its rapid setting characteristics.

### 5.9 Swelling test

Dried hydrogel samples (W_d_) were prepared by freeze-drying. Samples were immersed in deionized water at 25 °C until equilibrium swelling were reached. At timed intervals, samples were taken out, surface water was gently blotted, and the swollen weight (W_s_) were recorded. The equilibrium swelling ratio (SR) was calculated as:

SR = (W_s_ - W_d_) / W_d_

Measurements were performed in triplicate.

### 5.10 Oxygen release of MPO hydrogels

Ultrapure water was purged with nitrogen gas to remove dissolved oxygen before use. Then, 1 mL of MPO gel (235 mM) was mixed into 7 mL of water. At varied time points, the dissolved oxygen content of the solution was monitored using a portable dissolved oxygen meter.

### 5.11 Mg^2+^ release of MPO hydrogels

Mg^2+^ release was monitored using Slide-A-Lyzer MINI dialysis devices (Thermo Fisher, 3.5K MWCO). Typically, freshly prepared gel (approximately 25 μL, 6 μmol) was added to the dialysis chamber containing 1 mL of ultrapure water. The chamber was then placed into a conical tube containing 14 mL of Tris-HCl buffer (pH 5.5). The setup was gently shaken in the dark. At predetermined time points, 100 μL samples were taken from the conical tube after a thorough mixing, followed with the addition of an equal volume (100 μL) of corresponding buffer. Magnesium content of samples was then analyzed using ICP-OES.

### 5.12 H_2_O_2_ release of MPO hydrogels

H_2_O_2_ release was monitored using Slide-A-Lyzer MINI dialysis devices (Thermo Fisher, 3.5K MWCO). Typically, freshly prepared gel (approximately 25 μL, 6 μmol) was added to the dialysis chamber containing 1 mL of ultrapure water. The chamber was then placed into a conical tube containing 14 mL of Tris-HCl buffer with varied pH values. The setup was gently shaken in the dark. At preset time points, 300 μL samples were taken from the conical tube after a thorough mixing, followed with the addition of an equal volume (300 μL) of corresponding buffer. H_2_O_2_ concentrations of samples were then measured using the cerium sulfate method above-mentioned. To investigate H_2_O_2_ release profile in simulated physiological fluids, the chamber was placed into a conical tube containing 14 mL of simulating media containing 3 % serum proteins or lysozyme (0.5 mg/mL).

To investigate the H_2_O_2_ release of MPO under different state, diluted MPO solution was prepared by dissolving 250 mg of MPO hydrogel in 1.25 mL deionized water. The diluted MPO solution or intact MPO hydrogel was added to the dialysis chamber, respectively. The chambers were then placed into the conical tubes containing Tris-HCl buffer. At varied time points, samples were taken from the conical tube, and H_2_O_2_ concentrations were measured using the cerium sulfate method above-mentioned.

### 5.13 Neutralizing efficiency evaluation

200 μL of MPO hydrogel or ascorbic acid with varied concentrations were added into 4 % sodium hypochlorite aqueous solutions, and the final concentration of the neutralizing agents in the system became 2, 5, 8, 10, or 20 μM, respectively. The reaction was allowed to proceed for 2 h at room temperature. After that, the remaining oxidative products of the samples were measured to calculate the neutralization efficiency of the neutralizing agents.

### 5.14 Cell viability

HACAT, HUVEC, HDF cells were seeded in 24-well plates at a density of 5 × 10^4^ cells per well, respectively. After the cells got adhered, the medium was replaced with serum-free culture medium. Subsequently, transwell inserts containing 2 mM of MPO gel were placed into the wells and incubated at 37 °C for another 24 h. After that, cell proliferation was evaluated by CCK8 kit. As the comparison groups, the medium was replaced with 1 mL of serum-free medium containing the following agents: control, MgCl_2_ (2 mM), and H_2_O_2_ (2 mM), respectively.

Besides, to evaluate the neutralizing effect of the gel on oxidative chemicals, gels were premixed with sodium hypochlorite (3 μmol) and then placed in the transwell inserts before the incubation. As the control, sodium hypochlorite dispersed in alginate hydrogels was co-incubated with the cells prior to the assessment.

### 5.15 Tube formation

HUVEC cells were seeded onto Matrigel-coated 24-well plates (Corning) at a density of 1 × 10^5^ cells per well. After the cells adhered, the culture medium was replaced with 400 μL of serum-free medium, and transwell inserts containing different treatments - control, MgCl_2_ (0.7 mM), H_2_O_2_ (0.7 mM), and MPO gel (0.7 mM) - were then placed into the wells. The cells were co-cultured under standard conditions (37 °C, 5 % CO_2_) for 4 h. Following the incubation, tube formation was observed under a microscope. Quantitative analysis of angiogenesis was performed using ImageJ's Angiogenesis Analyzer module, measuring parameters such as nodes, junctions, and total vessel length.

### 5.16 Scratch assay

HUVEC cells were seeded in 6-well plates at a density of 2 × 10^5^ cells per well. Once the cells reached approximately 90 % confluence monolayer, a scratch was made using a pipette tip to create a uniform wound. The cells were then washed with PBS to remove any detached cells. The assay was conducted under serum-free conditions for all groups, and transwell inserts containing different treatments were then placed into the wells. Specifically, the Mg group was treated with serum-free medium containing 5 mM Mg^2+^, the H_2_O_2_ group with serum-free medium containing 1 mM H_2_O_2_, and the MPO group containing 5 mM MPO gel. The cells were incubated at 37 °C with 5 % CO_2_, and images of the scratch were captured at 0 and 24 h post-scratch. The wound healing rate was calculated based on the closure of the scratch over time.

### 5.17 Transwell migration assay

The cell migration assay was conducted using a Transwell migration assay to assess the migratory potential of HUVEC cells across different treatment groups: control, Mg^2+^ (0.7 mM), H_2_O_2_ (0.7 mM) and MPO (0.7 mM). HUVEC cells were seeded in the upper chamber of the Transwell inserts using 24-well plates, and serum-free medium was used in both the upper and lower chambers. The lower chamber contained different treatments. After incubation for 24 h under standard conditions (37 °C, 5 % CO_2_), the cells that had migrated to the lower surface of the insert were fixed, stained, and quantified to determine the migration rate.

### 5.18 RNA sequencing

HUVEC cells were seeded into 6-well plates at a density of 2 × 10^5^ cells per well. After cells achieved full adhesion, transwell inserts containing MPO hydrogel at a concentration of 0.5 mM (n = 3 wells per group) or untreated control inserts were placed into the wells. Cells were then co-cultured with the inserts for 24 h under standard conditions. Following the incubation period, cells were harvested, washed with cold phosphate-buffered saline, and immediately frozen at −80 °C for subsequent analysis. Total RNA was extracted using a commercial kit according to the manufacturer's instructions. RNA concentration and purity were assessed by a NanoDrop, and RNA integrity was verified by agarose gel electrophoresis. High-quality RNA samples were then used for cDNA library preparation, followed by next-generation sequencing on an appropriate platform. Raw sequencing data were processed and analyzed using standard bioinformatics pipelines for transcriptome profiling, including quality control, read alignment, gene expression quantification, and differential expression analysis.

### 5.19 WB analysis

HUVEC cells were seeded in 6-well plates at a density of 2 × 10^5^ cells per well. After achieving full adhesion, transwell inserts with MPO hydrogel at 0.5 mM and 1 mM (n = 3 wells/group) or untreated controls were introduced. Cells were cultured for 24 h, then harvested and lysed using RIPA buffer supplemented with protease inhibitors. Total protein concentrations were quantified via BCA assay. Equal amounts of protein were resolved by SDS-PAGE, transferred to PVDF membranes, and probed with primary antibodies against VEGF, FGF-2, ERK, p-ERK and HO-1 overnight at 4 °C, respectively. Membranes were subsequently incubated with HRP-conjugated secondary antibodies and visualized by chemiluminescence. GAPDH served as the loading control.

### 5.20 Biosafety assessment of MPO hydrogels *in vivo*

All animal experiments were approved by the Animal Experimentation Ethics Committee of Shanghai Jiao Tong University (Approval Code: A2025176). Female Kunming mice (8 weeks old) were randomly divided to two groups, with four animals in each group. Mice were depilated to prepare the skin for topical application. Either physiological saline or MPO gel was administered to the depilated skin area every other day over a 12-day observation period. Following the treatment schedule, all animals were euthanized, and blood was collected for hematology and serum biochemistry analysis. Serum samples were further analyzed for TNF-α and IFN-γ levels using enzyme-linked immunosorbent assay (ELISA) following the manufacturer's instructions. Major organs, including the heart, liver, kidney, spleen, lung, as well as skin tissues from the application site, were harvested for histopathological evaluation. Tissues were fixed in 10 % neutral buffered formalin, dehydrated, embedded in paraffin, and sectioned for hematoxylin and eosin (H&E) staining.

### 5.21 *In vivo* evaluation of MPO hydrogels on chemical burn skin wound healing

Six-week-old female Balb/c mice were used to create a partial-thickness chemical burn wound model. First, mice were anesthetized using isoflurane, and the dorsal hair was shaved to expose the skin. Then, A 10 mm diameter filter paper was soaked with 50 μl of 10 % sodium hypochlorite solution and carefully applied to the skin for a duration of 15 min, which induced a chemical burn. After the removal of the filter paper, the covered skin became a red wound.

Following the creation of the chemical burn model, mice were randomly divided into three groups (n = 6 per group). The groups were treated with 100 μl of either physiological saline, MPO gel (100 mM), or 2 % ascorbic acid solution, applied topically to the wound site every other day over a period of 10 days. Throughout the experimental period, mouse weight and wound healing progress were monitored. On days 2 and 10 post-treatment, one mouse in each group was randomly selected and sacrificed, and tissues were collected from the wound area for iNOS and IL-10 immunofluorescence staining. On day 10, samples were further subjected to K10 and K14 immunofluorescence staining, CD31 and SMA double immunofluorescence staining, as well as HE staining and Masson's trichrome staining. Quantitative analysis of the histological features was performed using ImageJ software, providing a comprehensive assessment of the wound healing process.

For comparation with conventional irrigation solutions and common burn dressings, mice were randomly divided into four groups (n = 5 per group). The groups were treated with 100 μl of either physiological saline, sodium bicarbonate (5 %), Ag-based dressing, or HA hydrogel (3 %), applied topically to the wound site every other day over a period of 10 days. Throughout the experimental period, mouse weight and wound healing progress were monitored. On days 10, mice were sacrificed, and tissues were collected from the wound area for HE staining and Masson's trichrome staining, and further subjected to iNOS and IL-10 immunofluorescence staining.

### 5.22 Statistical analysis

All the quantification were from at least three samples in each group. Data were shown as means ± standard deviation (SD), with n as the number of samples, cells, or animals. One-way ANOVA tests were applied for multiple comparisons. Statistical analysis was processed through GraphPad Prism 9 software. P < 0.05 was considered statistically significant, with ∗ p < 0.05, ∗∗p < 0.01, ∗∗∗p < 0.001.

## CRediT authorship contribution statement

**Meng Zhang:** Writing – original draft, Visualization, Project administration, Methodology, Investigation, Conceptualization. **Wanting Hao:** Writing – original draft, Visualization, Software, Methodology, Investigation, Formal analysis, Data curation. **Zi Fu:** Writing – review & editing, Validation, Resources, Methodology, Investigation, Formal analysis, Data curation. **Ying Huang:** Software, Investigation. **Fuhua Yan:** Supervision, Resources, Funding acquisition. **Dalong Ni:** Writing – review & editing, Supervision, Resources, Project administration, Funding acquisition.

## Ethics approval and consent to participate

All animal experiments were approved by the Animal Experimentation Ethics Committee of Shanghai Jiao Tong University (Approval Code: A2025176), ensuring that all animal treatments adhered strictly to the National Institutes of Health's guidelines for the Care and Use of Laboratory Animals.

## Declaration of competing interest

The authors declare that they have no known competing financial interests or personal relationships that could have appeared to influence the work reported in this paper.
