# Supplementary material for: Self-assembled magnesium peroxide supramolecular hydrogel for oxidants neutralization and chemical burn management
Source: Bioact Mater. 2025 Nov 12;57:154–68. doi: 10.1016/j.bioactmat.2025.11.002 (PMC12657613; doi:10.1016/j.bioactmat.2025.11.002)
Supplement: Multimedia component 1 [file mmc1.docx]

**Supplementary Materials**

**Self-Assembled Magnesium Peroxide Supramolecular Hydrogel for Oxidants Neutralization and Chemical Burn Management**

**This PDF file includes:**

**Figure S1 to S39**


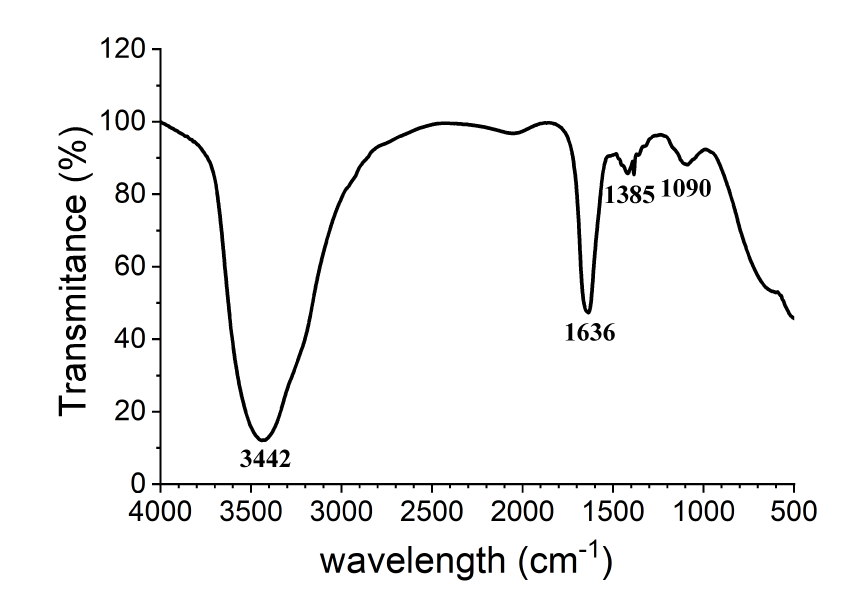


Figure S1: FT-IR pattern of freeze-dried MPO powder.


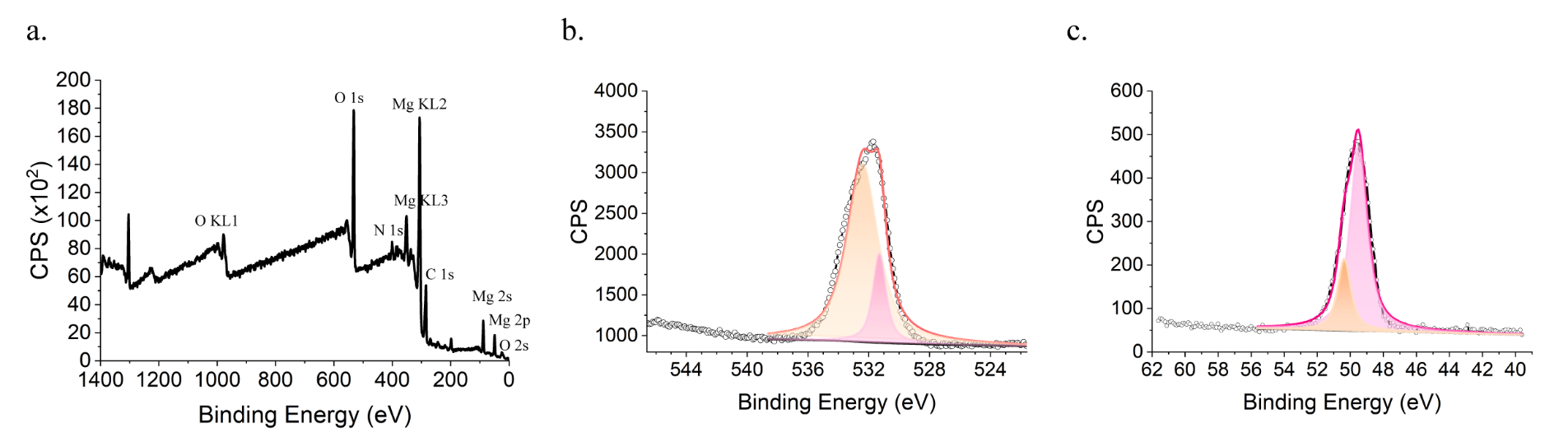


Figure S2: a. XPS survey scan of freeze-dried MPO powder. b. High-resolution O1s XPS spectra of MPO. c. High-resolution Mg2p XPS spectra of MPO.


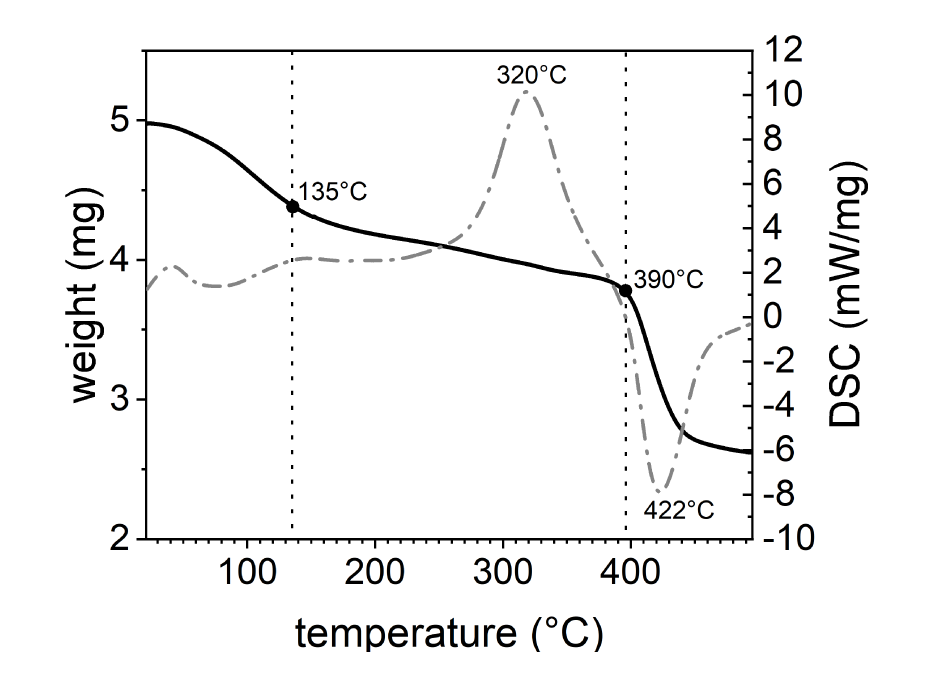


Figure S3: Simultaneous thermal analysis of freeze-dried MPO powder.


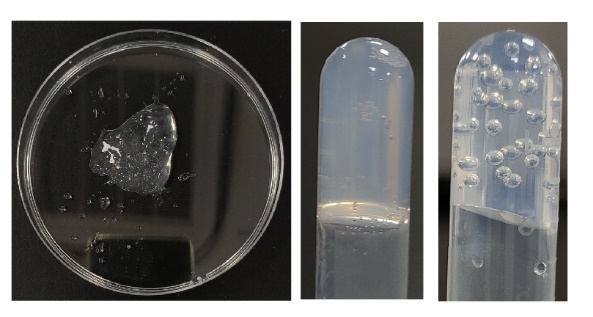


Figure S4: The transparent appearance of MPO hydrogels. When the hydrogel stands for 5 hours at room temperature, a number of bubbles generated.


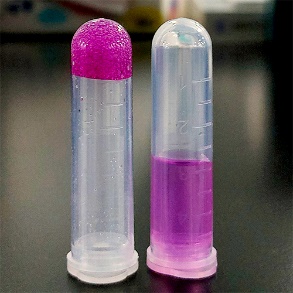


Figure S5: MPO products (with red dye) distributed in water with a higher concentration form the hydrogel (left), and with a lower concentration could not hydrogelation (right).


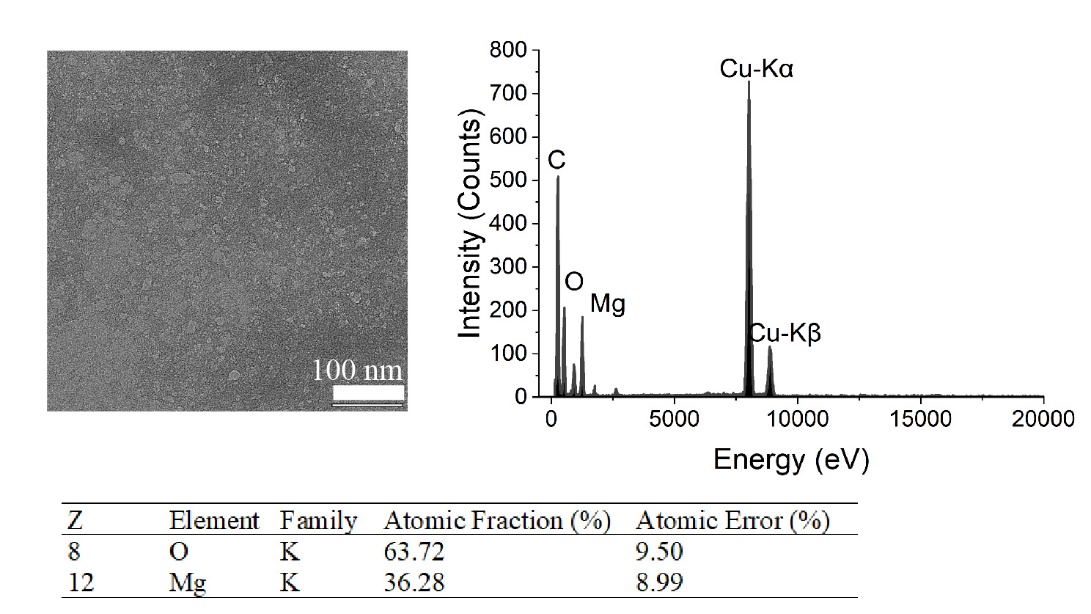


Figure S6: Energy-dispersive X-ray spectroscopy (EDS) analysis of MPO hydrogels.


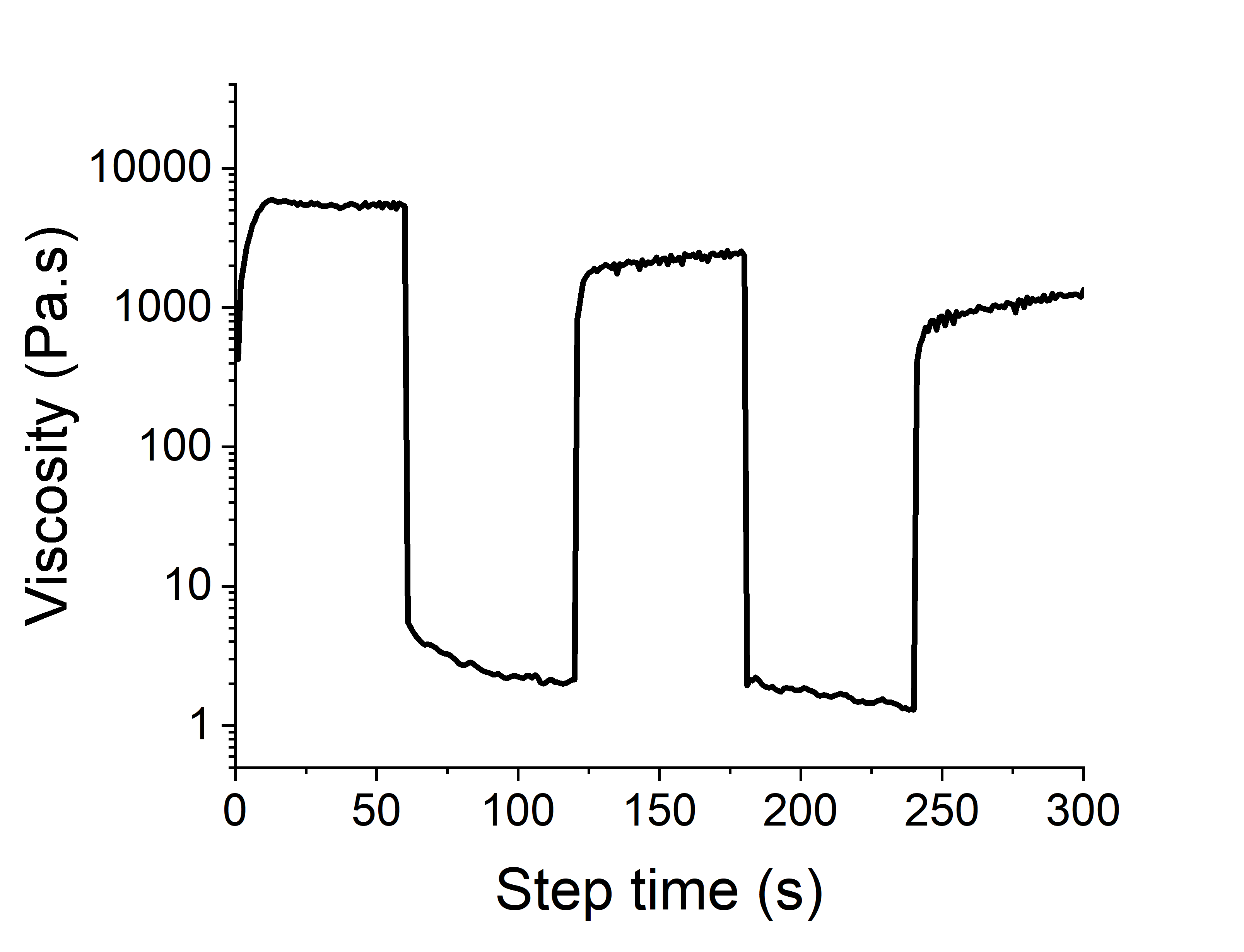


Figure S7: Oscillatory recovery tests of MPO hydrogel.


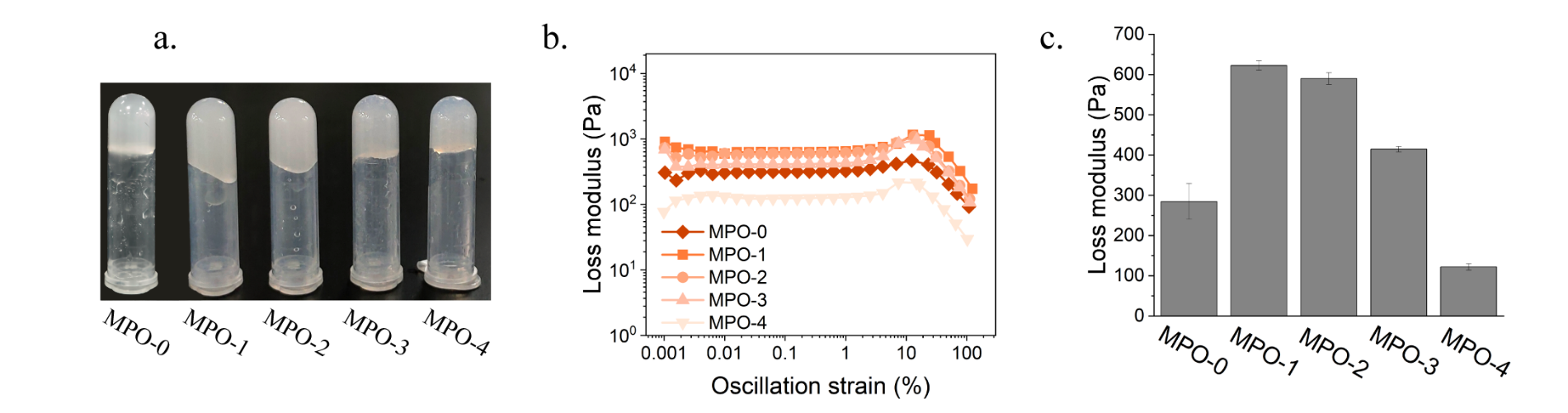


Figure S8: a, Hydrogel photos of MPO-0, MPO-1, MPO-2, MPO-3 and MPO-4. b, Plots of G’’ (loss moduli) versus oscillation strain for MPO-0, MPO-1, MPO-2, MPO-3 and MPO-4. c, G’’ value of MPO-0, MPO-1, MPO-2, MPO-3 and MPO-4 at 0.1% of oscillation strain.


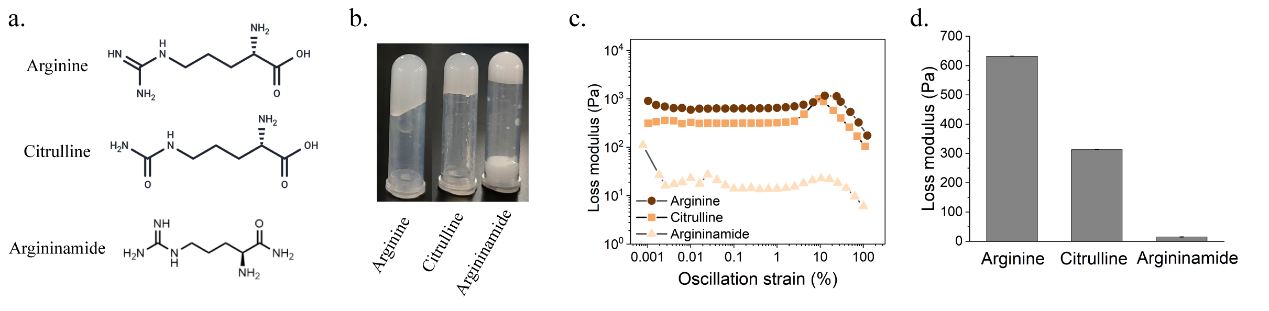


Figure S9: a, Chemical structures of arginine, citrulline and argininamide, respectively. b, Hydrogel photos prepared with arginine (MPO-1), citrulline and argininamide. c, Plots of G’’ (loss moduli) versus oscillation strain for MPO hydrogels prepared with arginine, citrulline and argininamide. d, G’’ value of MPO hydrogels prepared with arginine, citrulline and argininamide at 0.1% of oscillation strain.


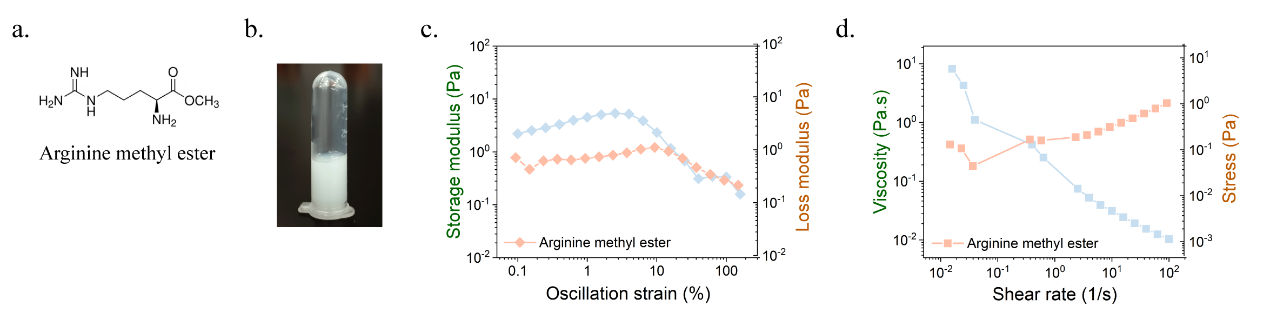


Figure S10: a, Chemical structures of arginine methyl ester. b, Photo of the MPO product prepared with arginine methyl ester. c, Plots of G’ and G’’ versus oscillation strain for MPO product prepared with arginine methyl ester. d, Plots of viscosity and stress versus shear rate for MPO product prepared with arginine methyl ester.


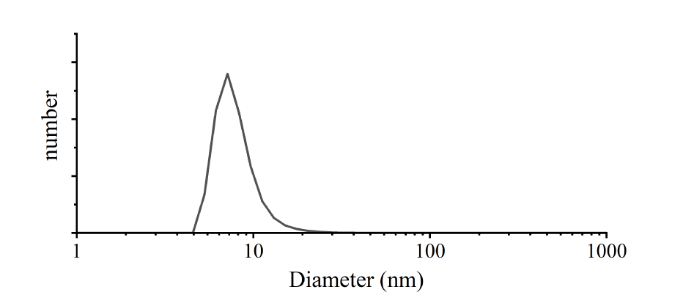


Figure S11: DLS analysis of diluted MPO-1 product in solution.


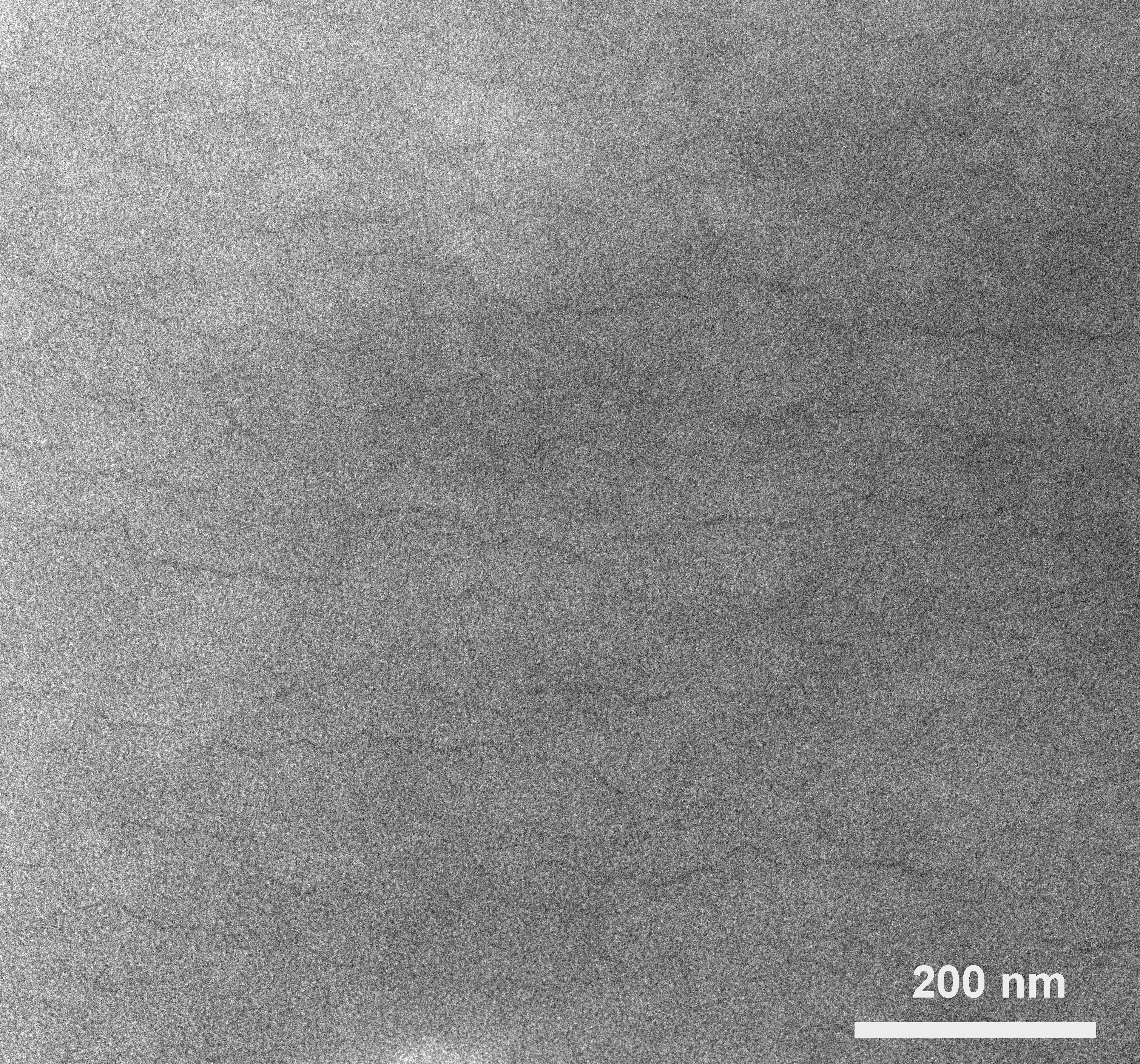

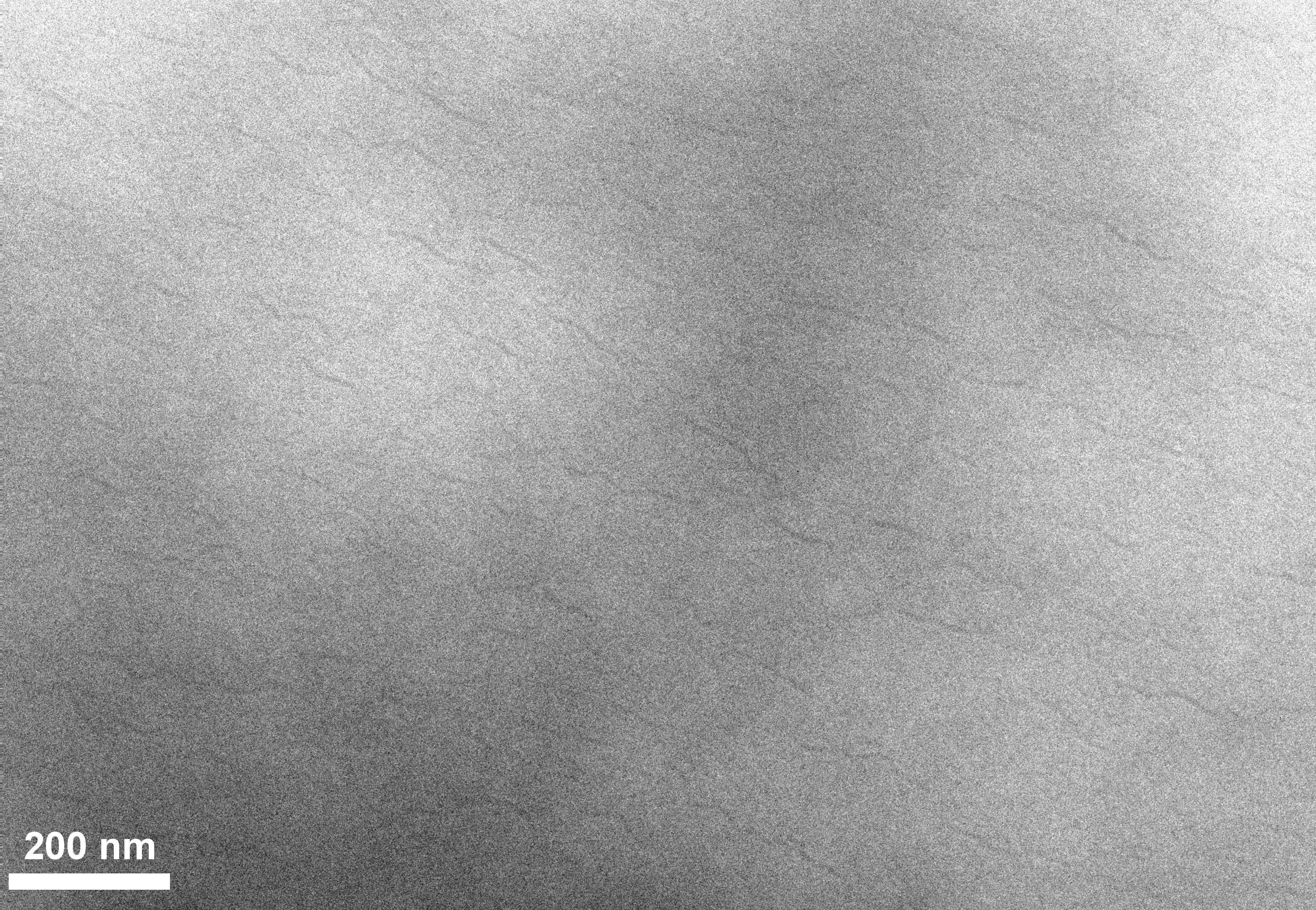


Figure S12: TEM images of MPO hydrogel.


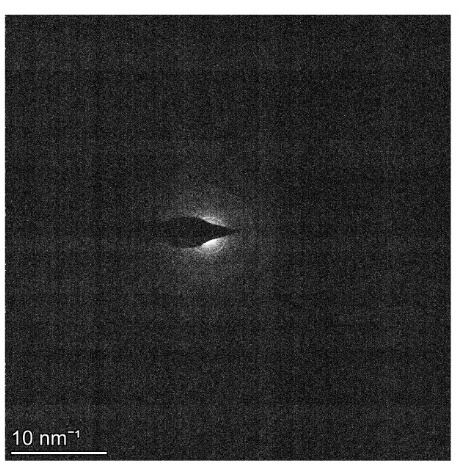


Figure S13: Selected area electron diffraction (SAED) analysis of MPO hydrogels.


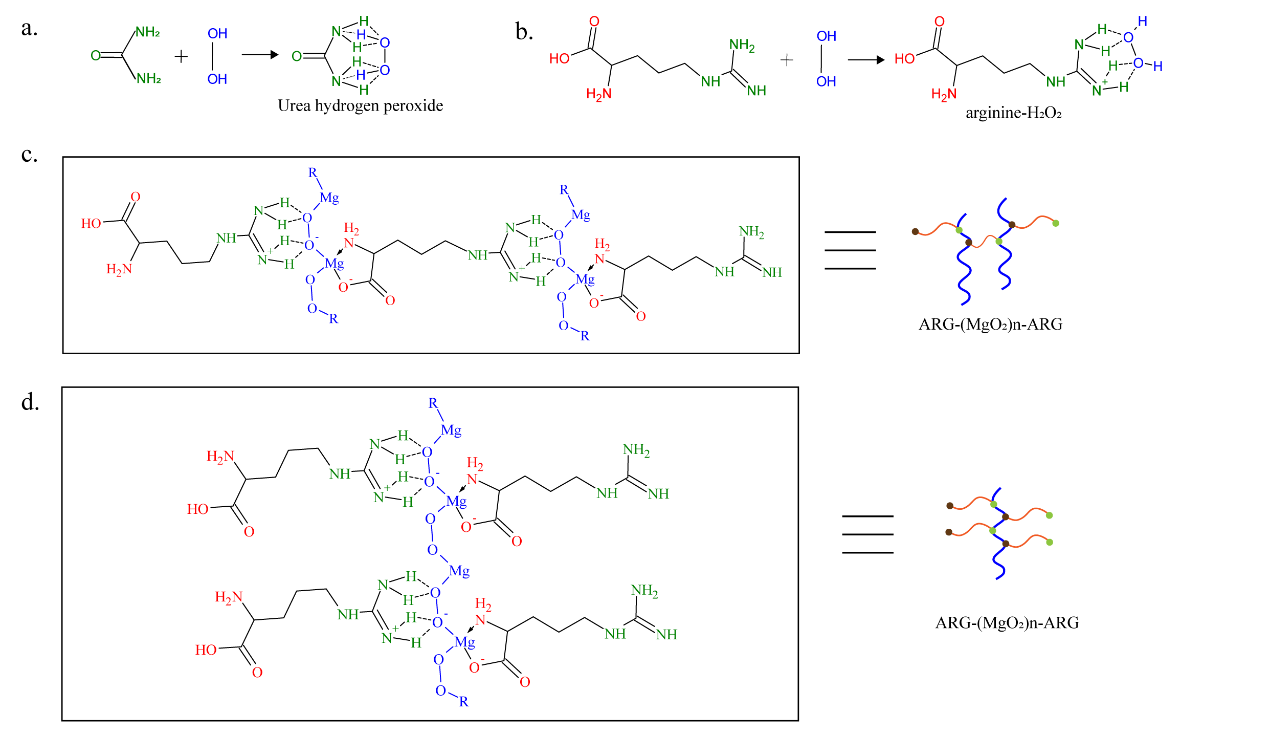


Figure S14: a, The formation of urea hydrogen peroxide. b, Plausible formation mechanism of arginine-hydrogen peroxide. c-d, Plausible formation mechanism of MPO hydrogels.


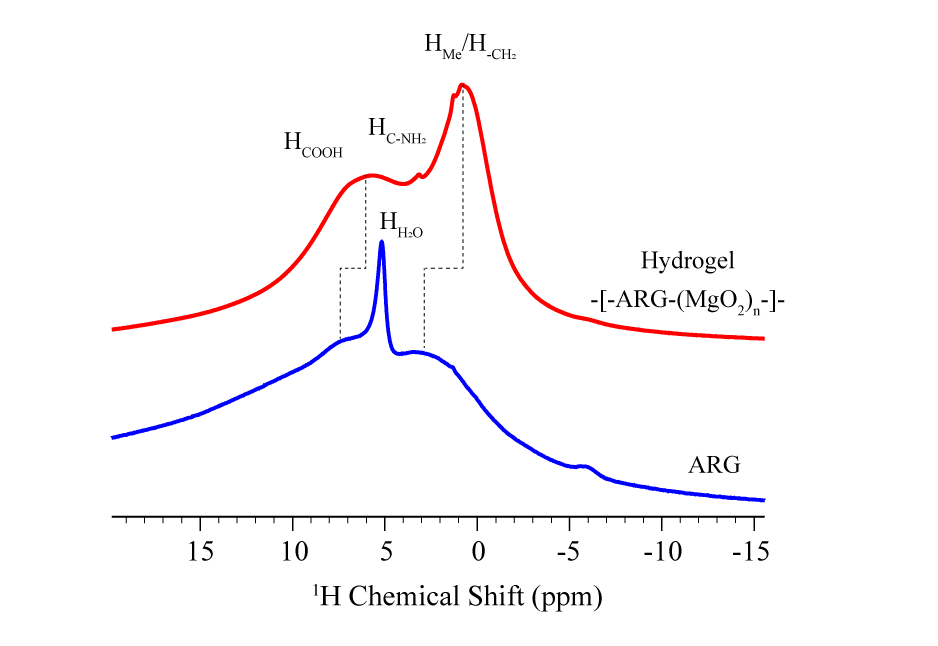


Figure S15: 1H MAS ssNMR spectra of MPO hydrogel and ARG.


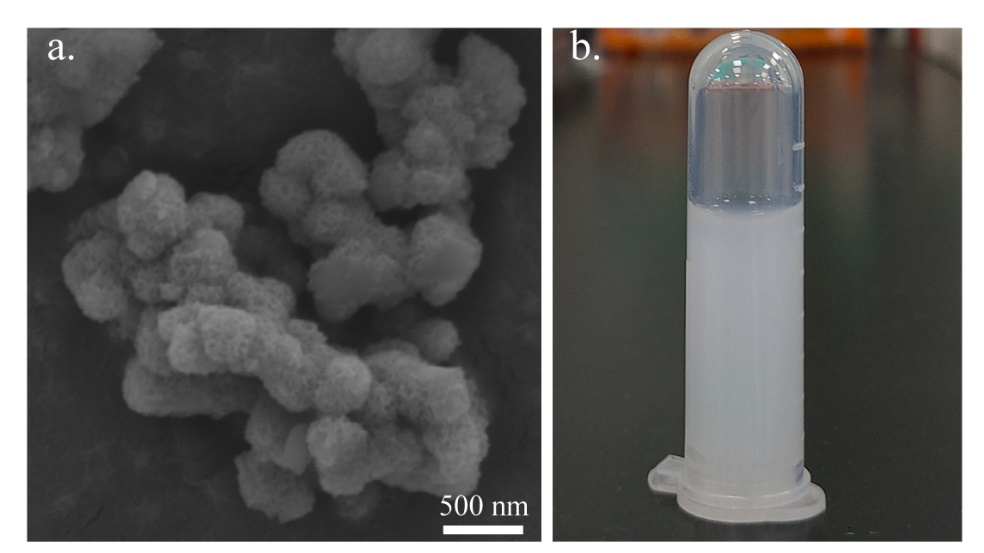


Figure S16: a. SEM images of the pre-synthesized MPO nanoparticles. b. Mixture of the pre-synthesized MPO nanoparticles solution and arginine solution.


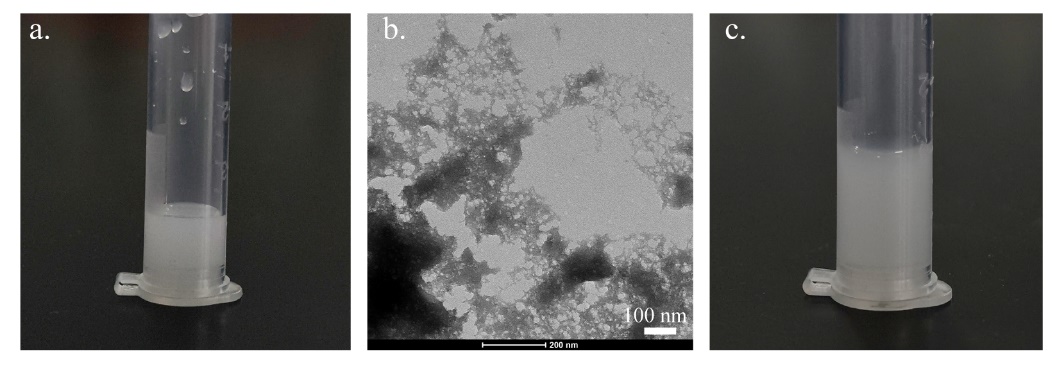


Figure S17: a. Photo of the MPO-5 product synthesized without the addition of arginine. b. TEM images of MPO-5 product synthesized without the addition of arginine. c. Photo of the mixture of MPO-5 solution and arginine. After the synthesis of MPO-5, arginine was added to the reaction system.


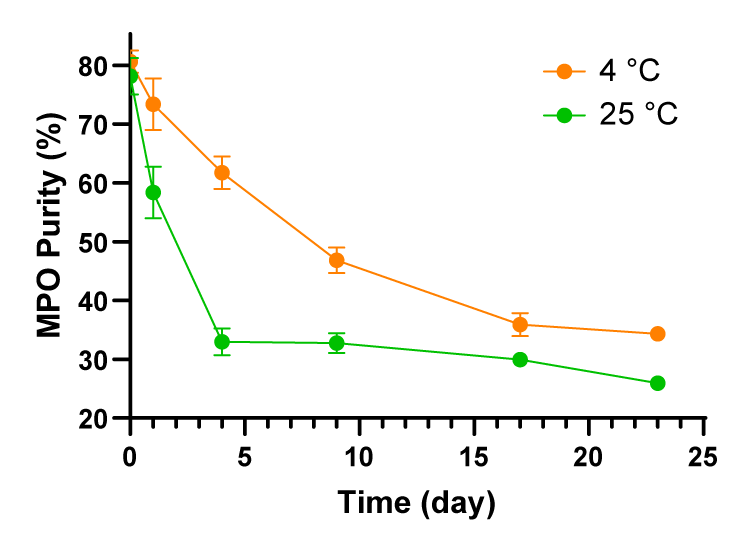


Figure S18: MPO purities of the hydrogel when stored at 4°C or 25°C.


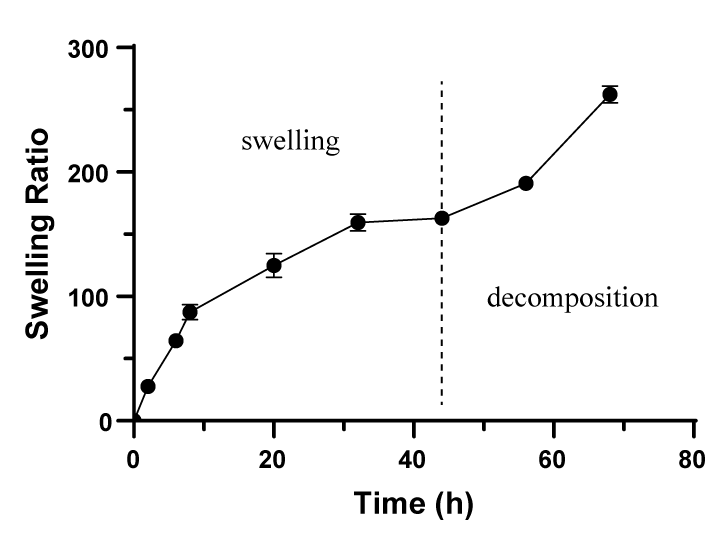


Figure S19: Swelling property of MPO hydrogel. The hydrogel reached swelling equilibrium point at 42 hours, after which the hydrogel could not maintain the gel form and showed as decomposition.


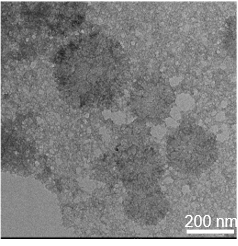


Figure S20: TEM images of MPO hydrogel at day 5 (magnified images, correspond to Figure 3b).


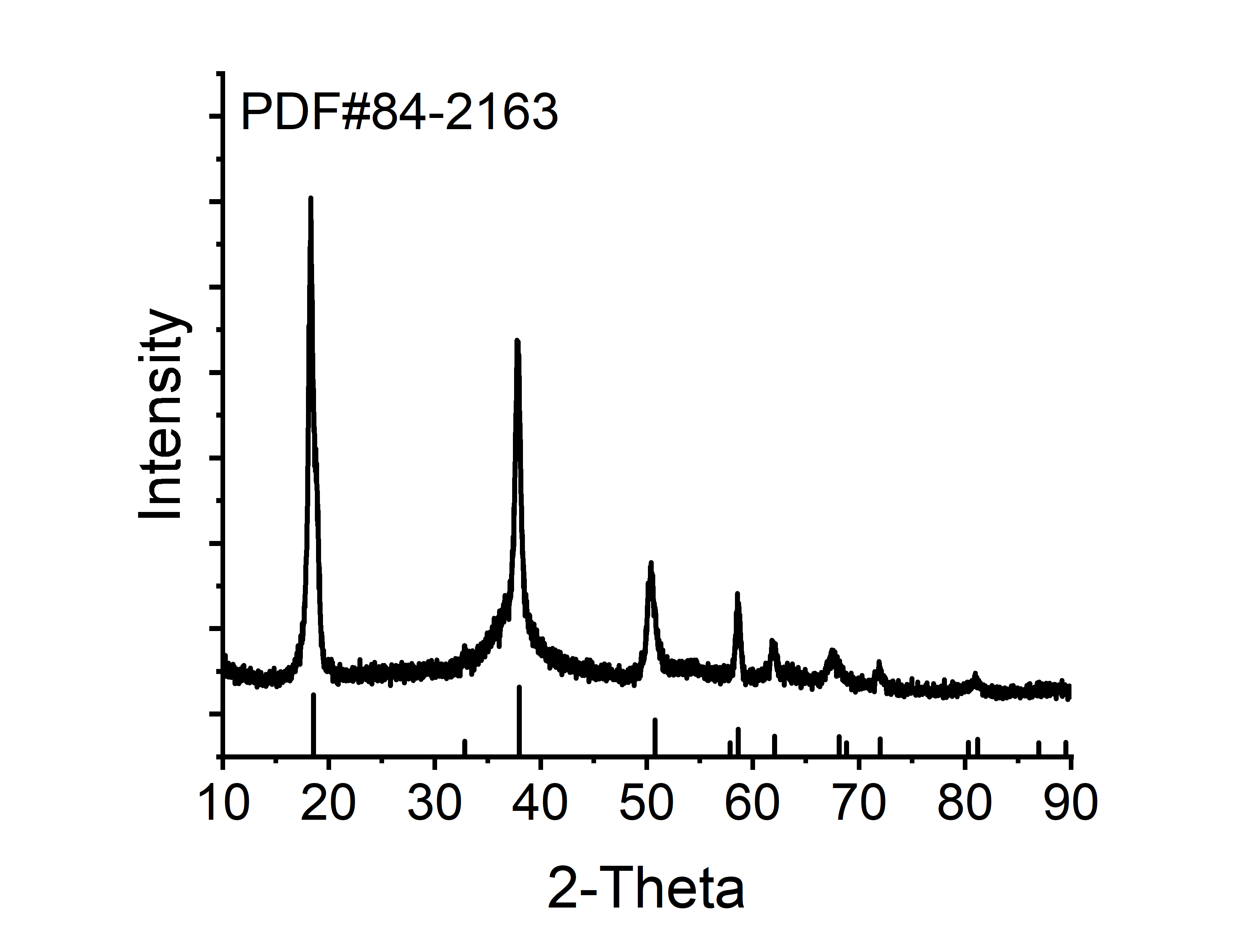


Figure S21: XRD pattern of MPO hydrogel after 15 days.


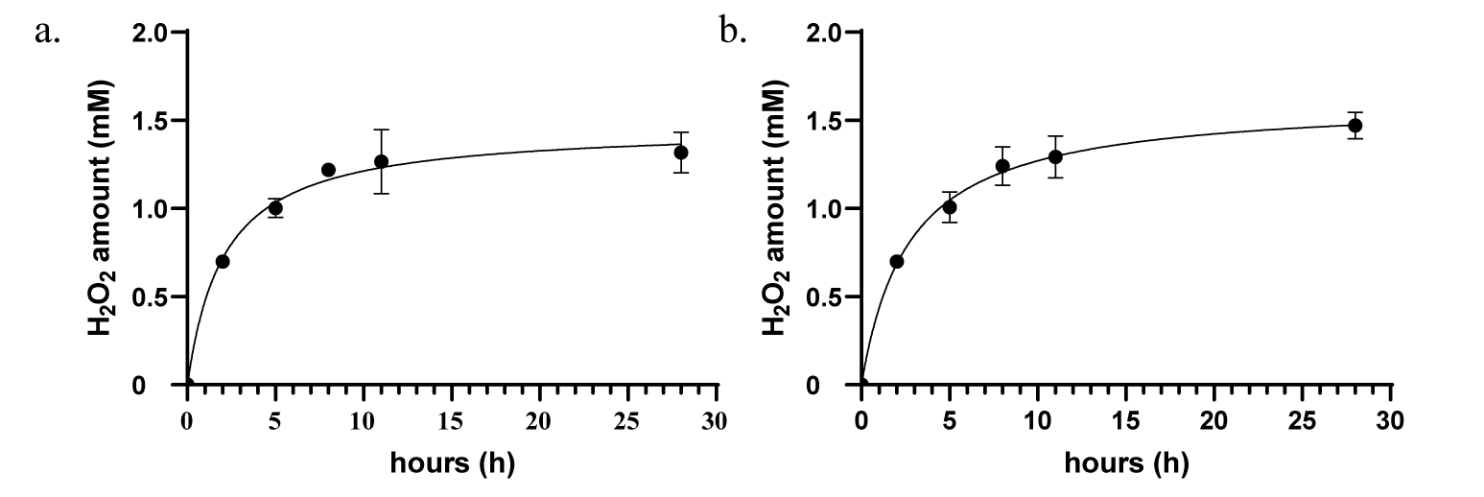


Figure S22: a. H_2_O_2_ release from MPO hydrogel in simulated physiological media containing 3% serum proteins. b. H_2_O_2_ release from MPO hydrogel in simulated physiological media containing lysozyme (0.5 mg/mL).


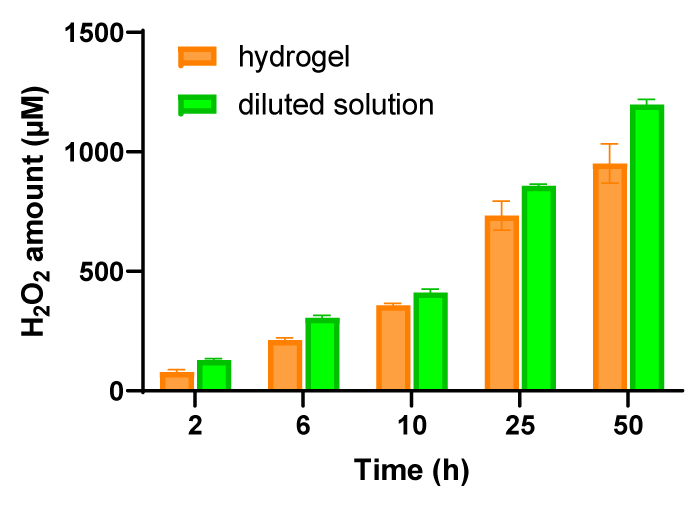


Figure S23: H_2_O_2_ release of MPO hydrogels and diluted MPO solution.


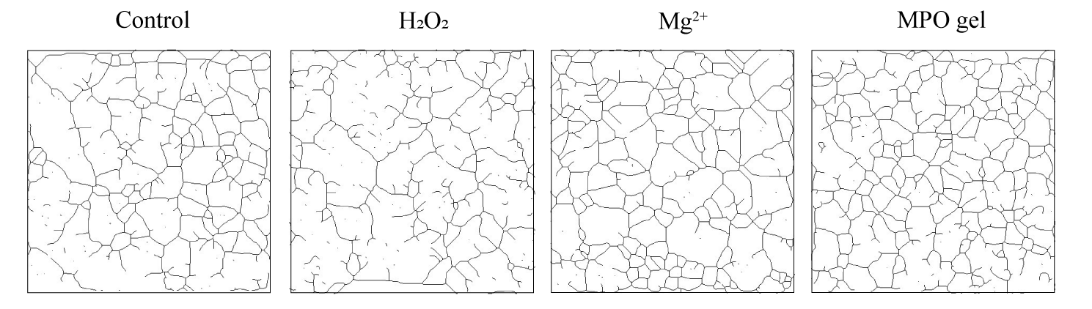


Figure S24: Binary maps of final binary pruned tree and elements of network for tube formation assay.


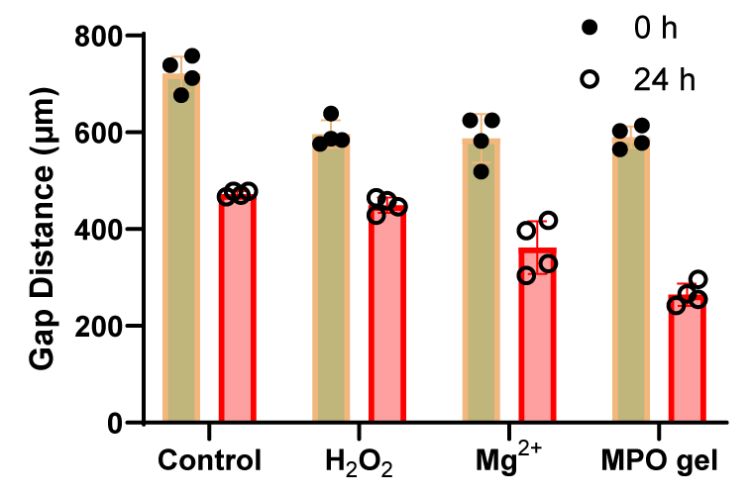


Figure S25: Quantification analysis of the gap distance of different groups in the wound scratch assay.


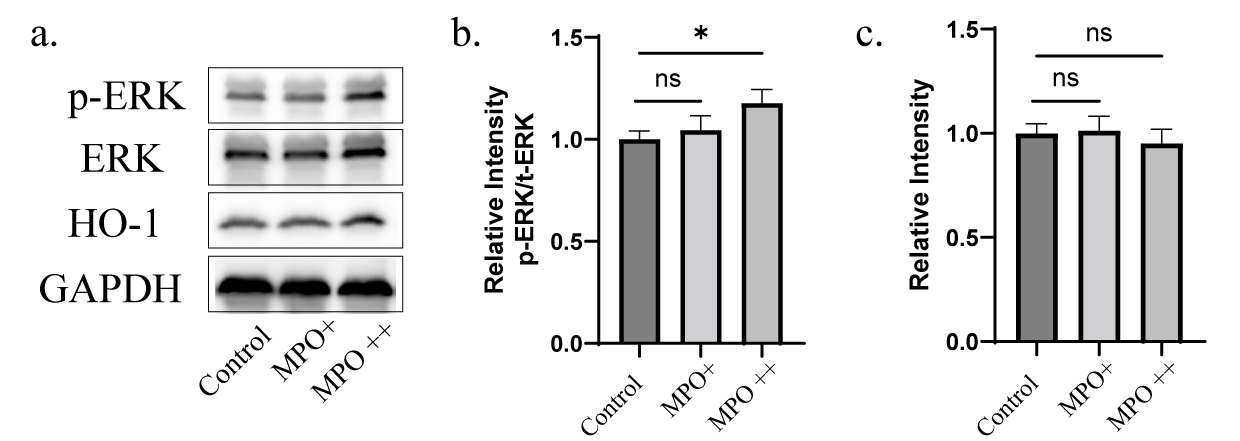


Figure S26: a. Western blotting in HEUVC cells after treated with different amount of hydrogel. b. Quantitative analysis of the relative levels of p-ERK/t-ERK. c. Quantification of the relative expression of HO-1 protein.


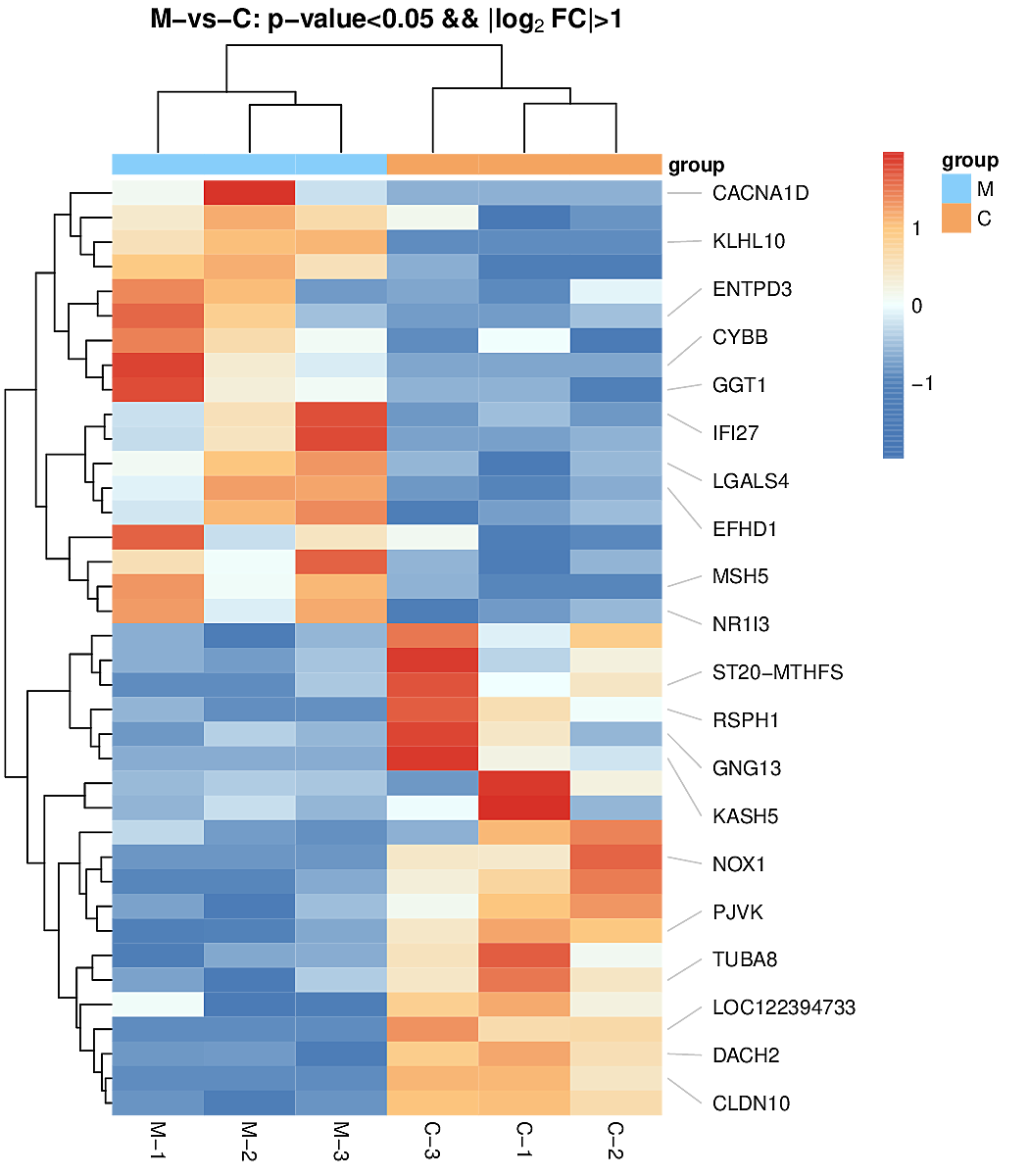

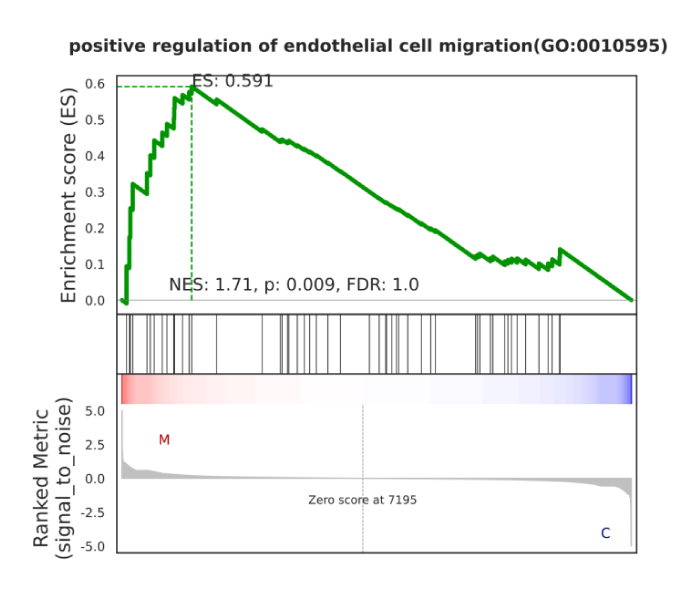


Figure S27: Heat map of differentially expressed genes between control group (C) and MPO treated group (M) (left), and GSEA analysis of the genes related to positive regulation of endothelial cell migration (right).


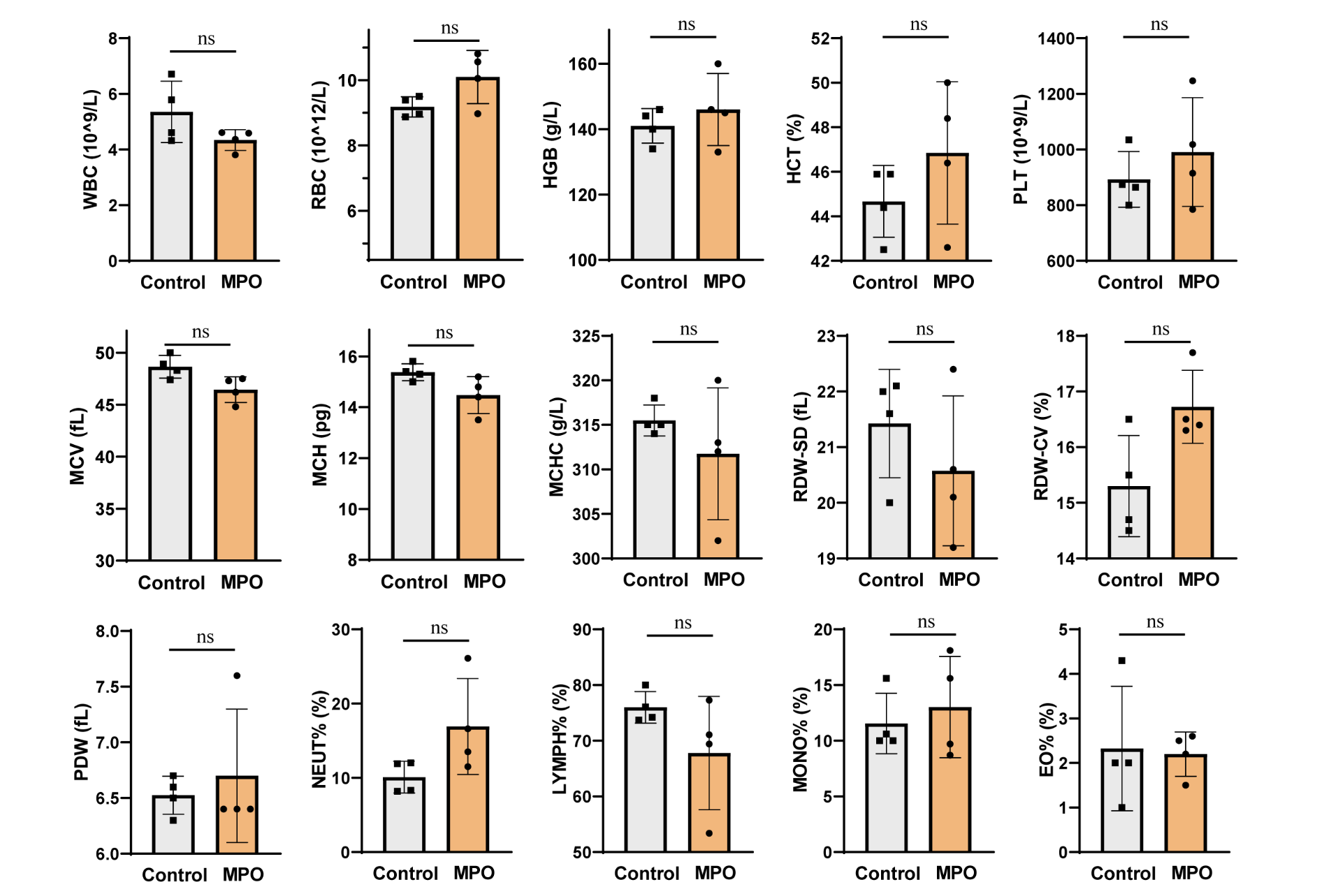


Figure S28: Hematology analysis of the blood with or without the treatment of MPO gels.


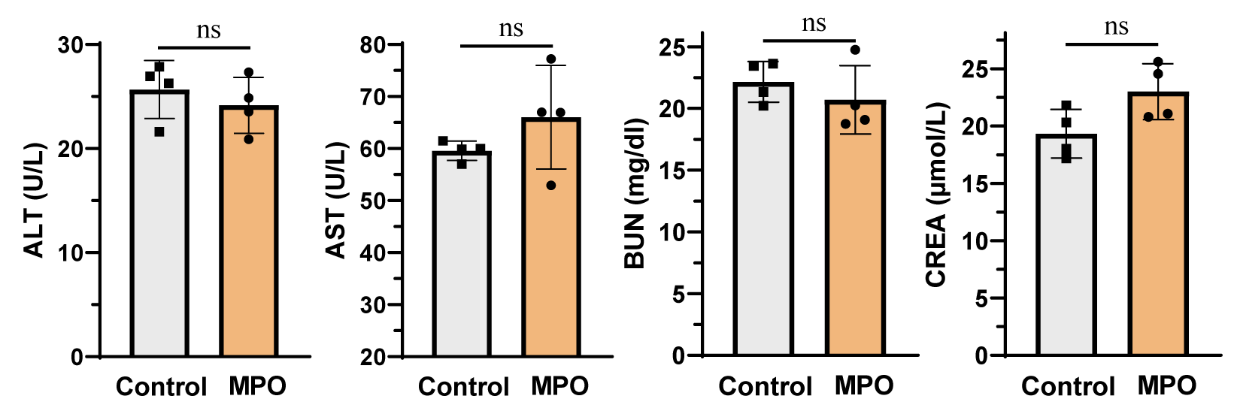


Figure S29: Serum biochemistry analysis of the blood with or without the treatment of MPO gels.


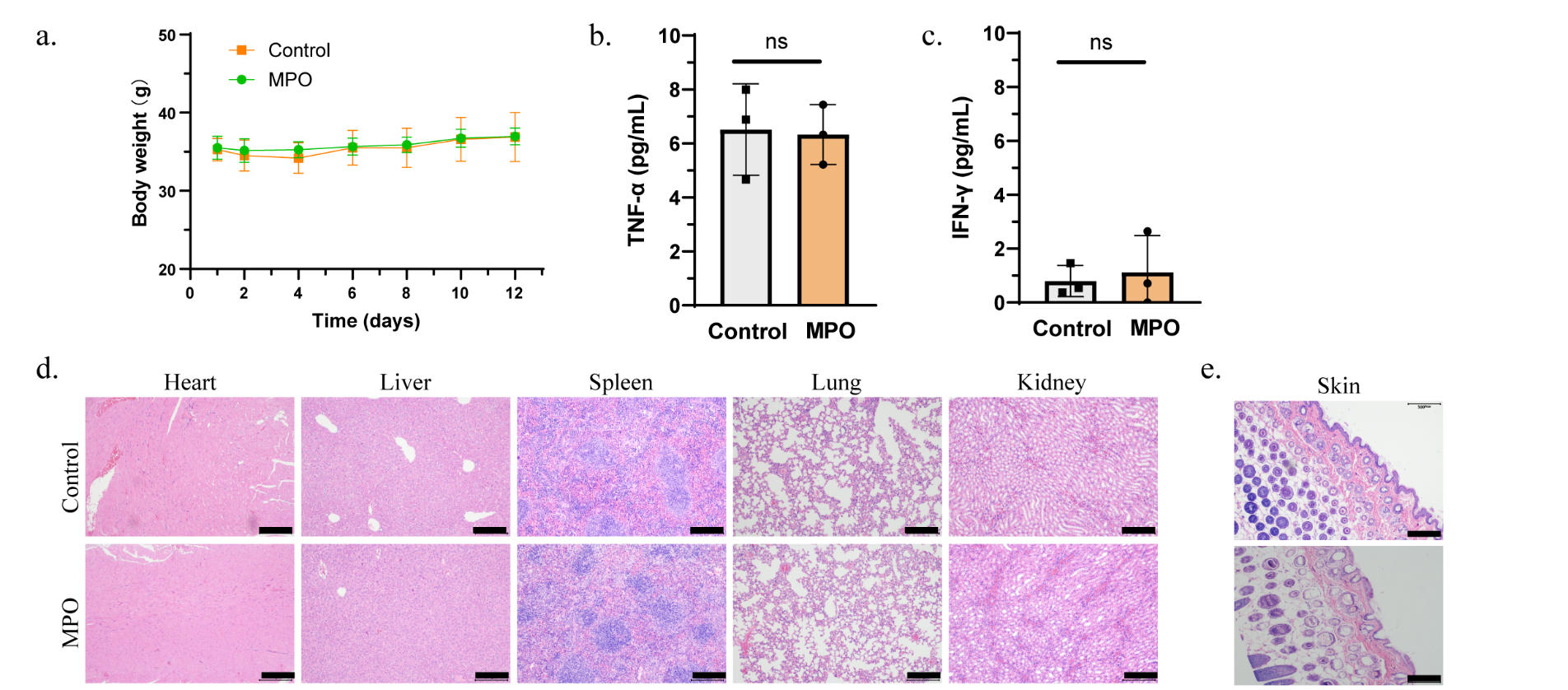


Figure S30: a. Body weight of mice after different treatment. b. TNF-α levels of serum samples analyzed by enzyme-linked immunosorbent assay (ELISA). c. IFN-γ levels of serum samples analyzed by enzyme-linked immunosorbent assay (ELISA). d. H&E staining of main organs after different treatment. Scale bar: 200 μm. e. H&E staining of skin tissues after different treatment. Scale bar: 200 μm.


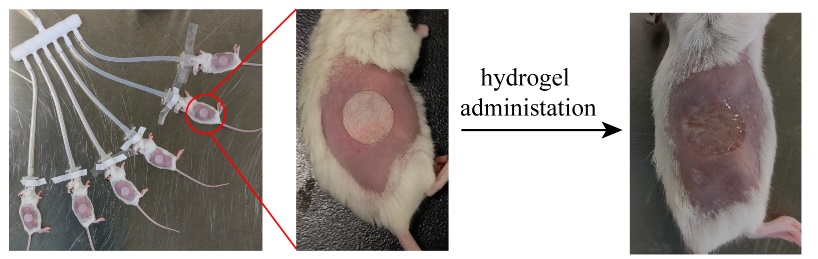


Figure S31: Images of the construction of the chemical burn wound model on mice, and the administration of MPO hydrogels.


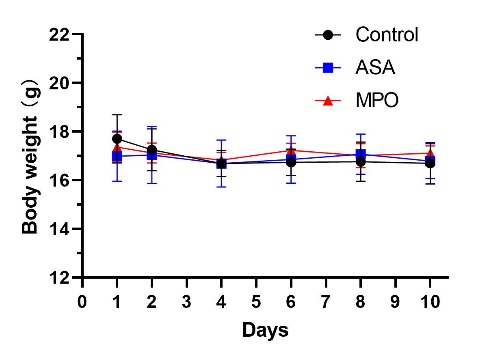


Figure S32: Body weight of mice after different treatment on chemical burn skin wound.


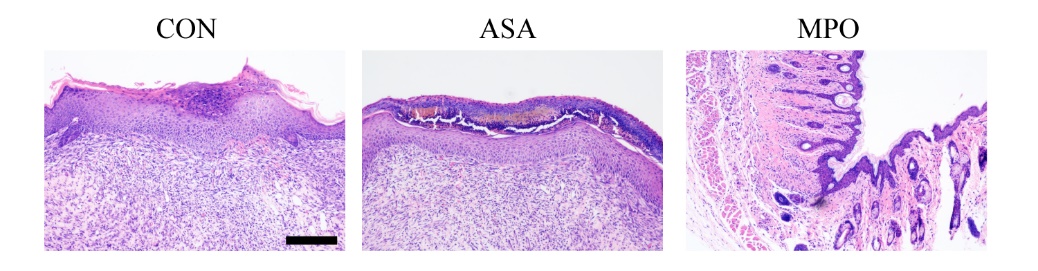


Figure S33: H&E staining of the skin wound tissues on day 10 after different treatment (magnified images, correspond to Figure 6e). Scale bar: 200 μm.


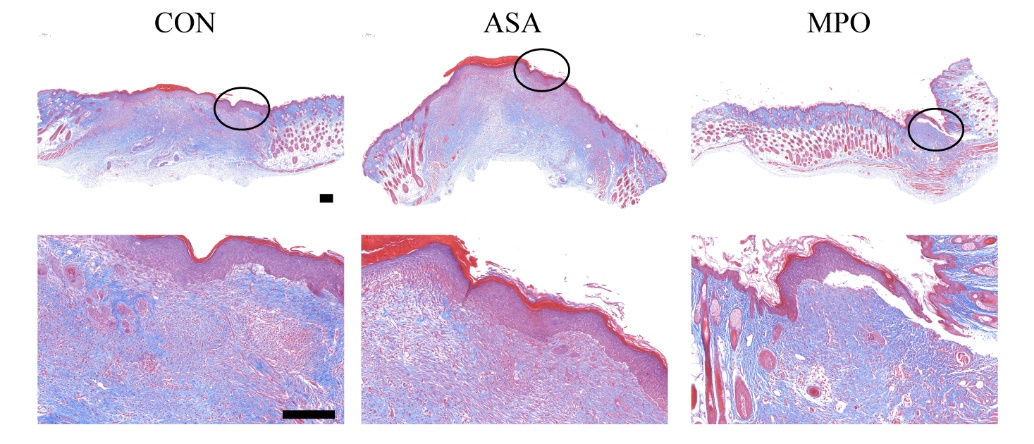


Figure S34: Masson staining of the skin wound tissues on day 10 after different treatment (general views and magnified images, correspond to Figure 6h). Scale bar: 100 μm.


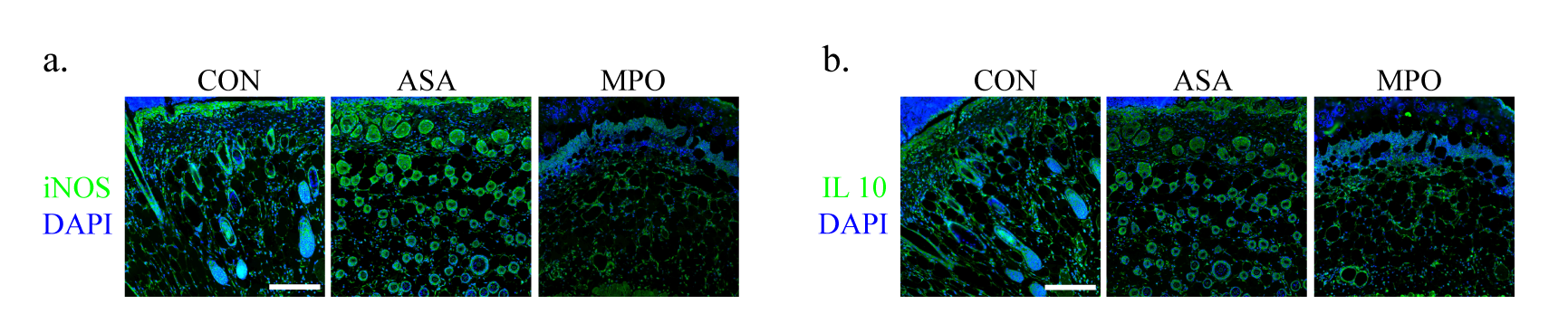


Figure S35: a. IF staining of iNOS at day 2 (magnified image, correspond to Figure 7a). Scale bar: 200 μm. b. IF staining of IL-10 at day 2 (magnified image, correspond to Figure 7a). Scale bar: 200 μm.


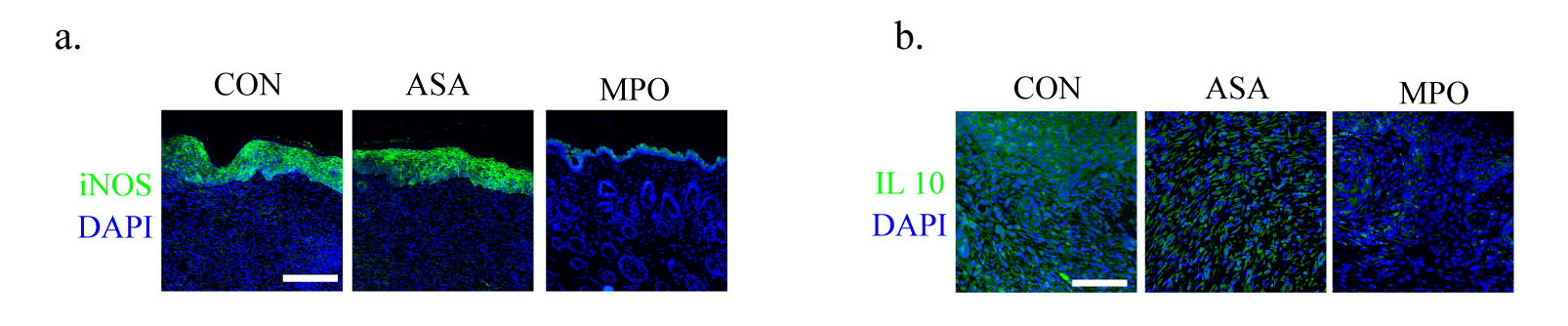


Figure S36: a. IF staining of iNOS at day 10 (magnified image, correspond to Figure 7b). Scale bar: 200 μm. b. IF staining of IL-10 at day 10 (magnified image, correspond to Figure 7b). Scale bar: 200 μm.


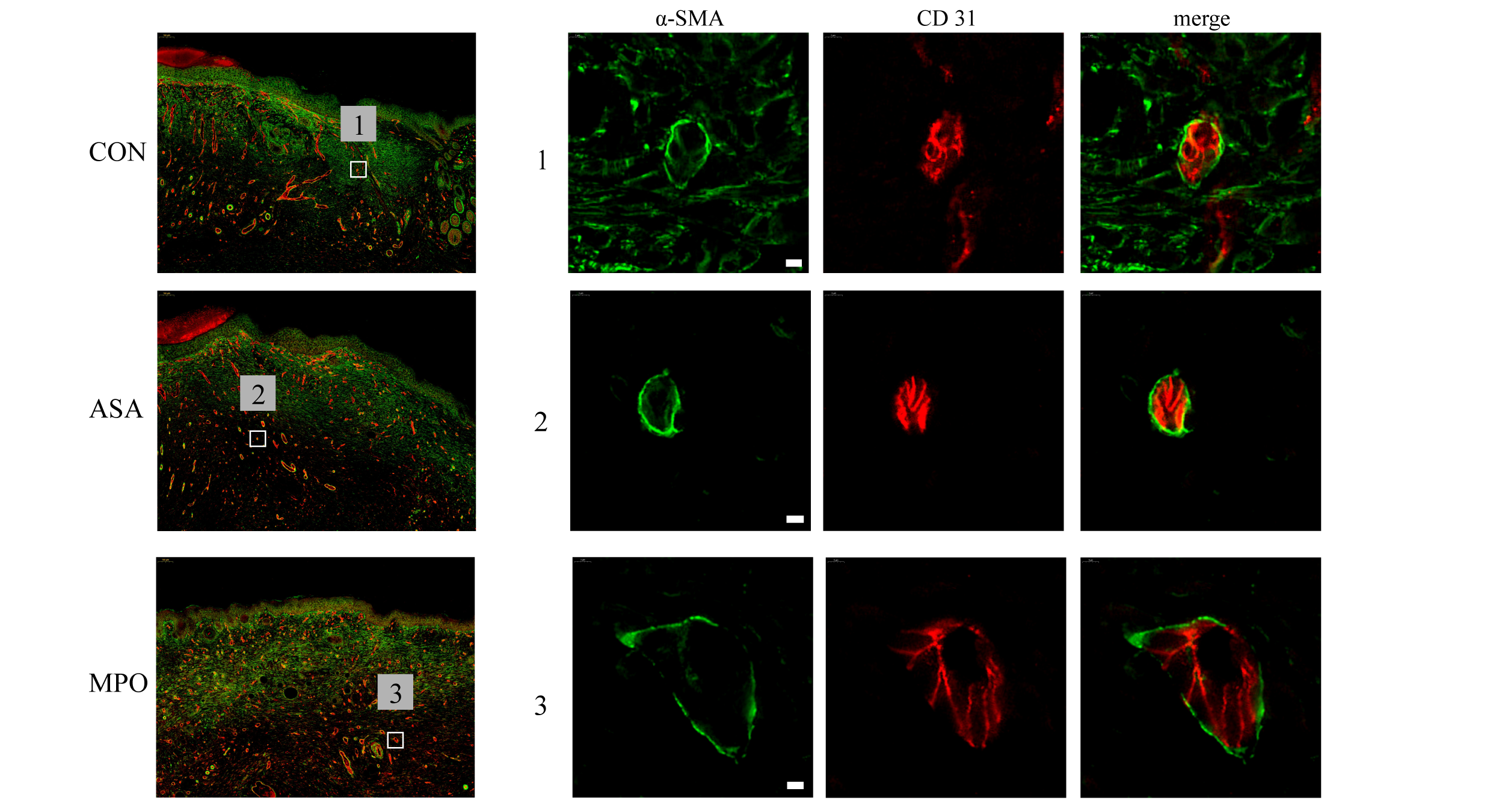


Figure S37: IF double staining of CD31 and α-SMA at day 10 (magnified image, correspond to Figure 7f). Scale bar: 5 μm.


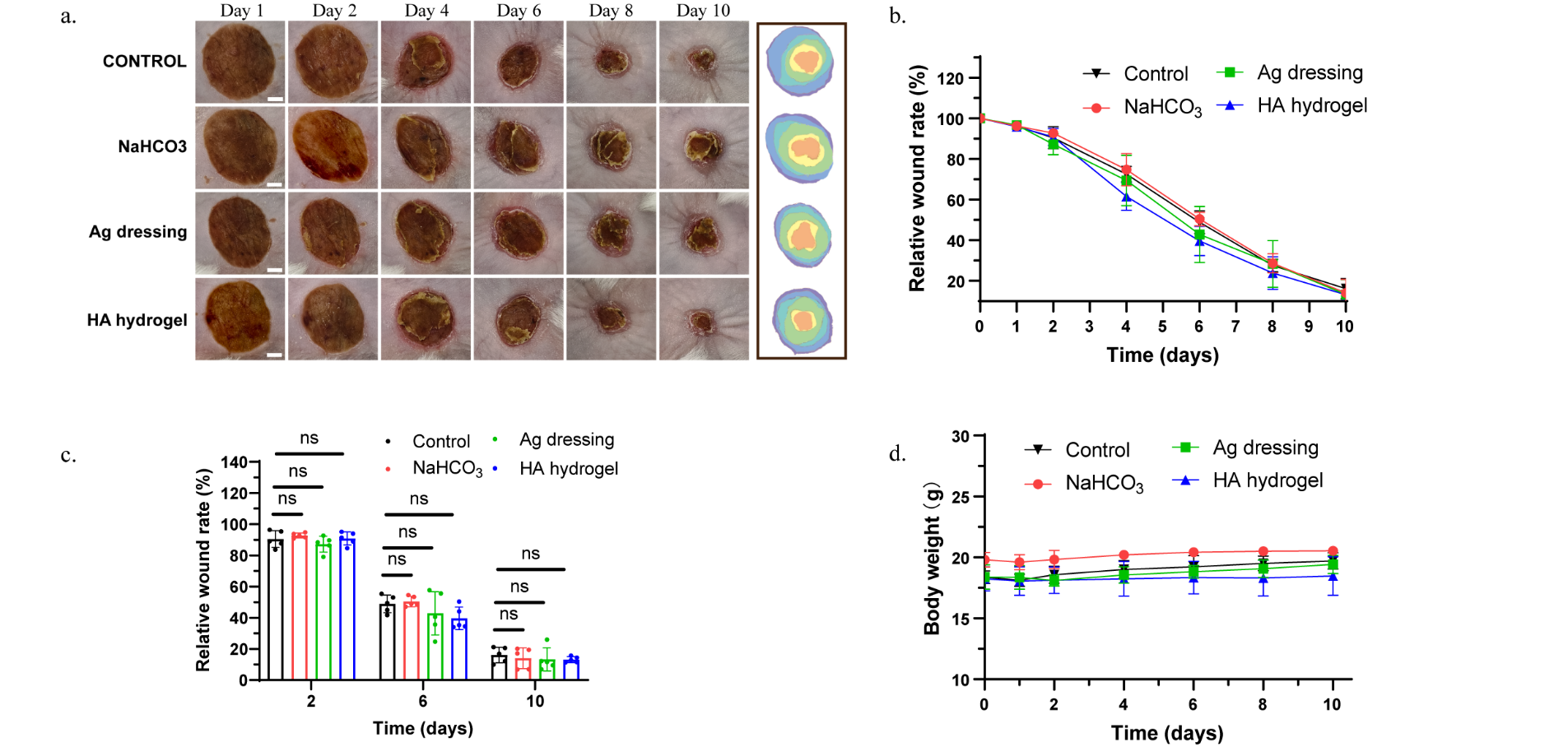


Figure S38: (a) Wound images after different treatment on chemical burn skin wound repair. Scale bar: 2 mm. (b) Wound closure rate after different treatment on chemical burn skin wound. (c) Relative wound area for different groups at day 2, 6 and 10. (d) Body weight of mice after different treatment on chemical burn skin wound. * p < 0.05, ** p < 0.01, *** p< 0.001, by one-way ANOVA tests.


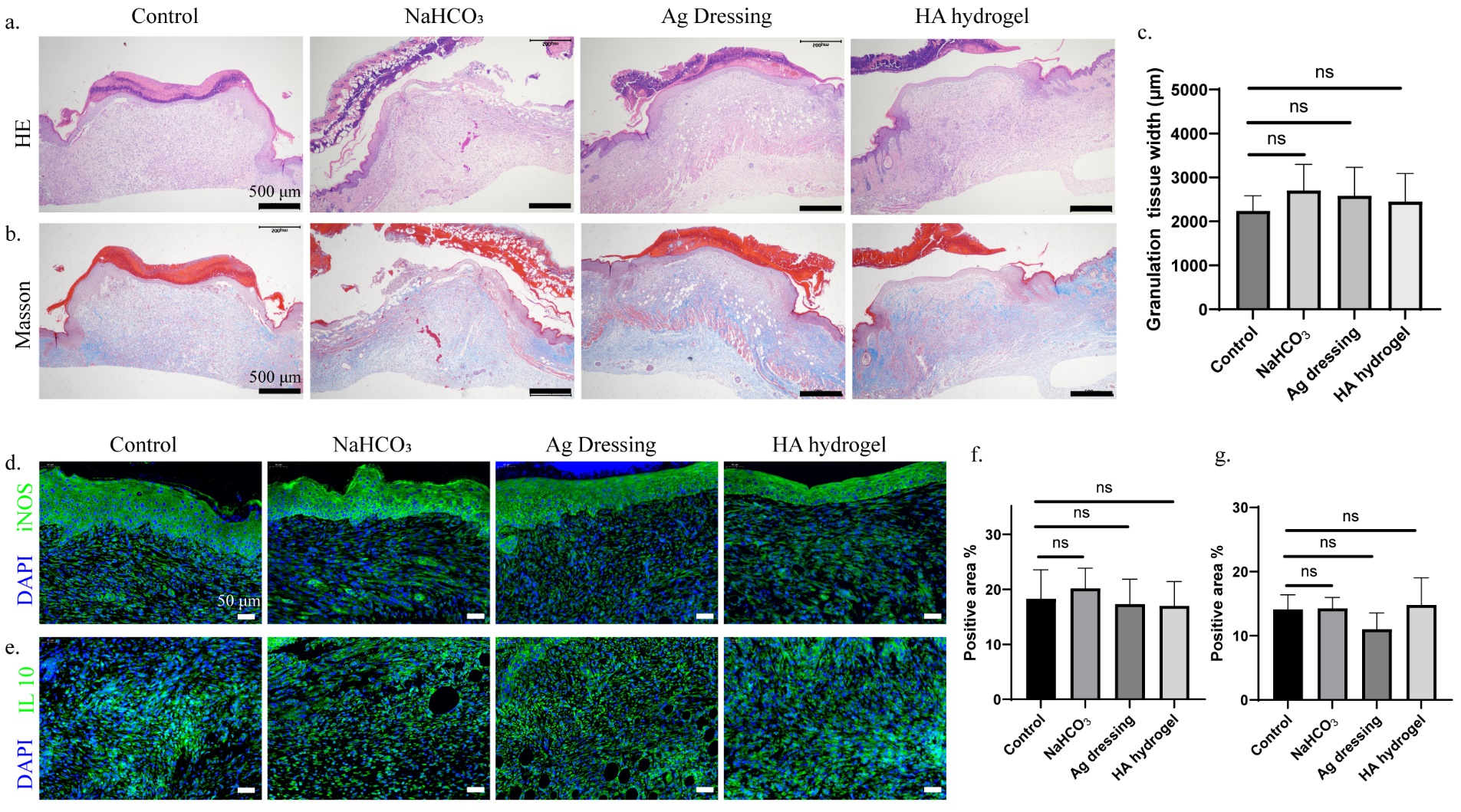


Figure S39: (a) H&E staining of the skin wound tissues on day 10 after different treatment. Scale bar: 500 μm. (b) Masson staining of the skin wound tissues on day 10 after different treatment. Scale bar: 500 μm. (c) Quantification analysis of the granulation tissue width of H&E staining. (d) IF staining of iNOS at day 10. Scale bar: 50 μm. (e) IF staining of IL-10 at day 10. Scale bar: 50 μm. (f) Quantification analysis of iNOS at day 10. (g) Quantification analysis of IL-10 at day 10. * p < 0.05, ** p < 0.01, *** p< 0.001, by one-way ANOVA tests.
